# Bojungikki-Tang Improves Response to PD-L1 Immunotherapy by Regulating the Tumor Microenvironment in MC38 Tumor-Bearing Mice

**DOI:** 10.3389/fphar.2022.901563

**Published:** 2022-07-06

**Authors:** Jaemoo Chun, Sang-Min Park, Jin-Mu Yi, In Jin Ha, Han Na Kang, Mi-Kyung Jeong

**Affiliations:** ^1^ KM Convergence Research Division, Korea Institute of Oriental Medicine, Daejeon, South Korea; ^2^ KM Data Division, Korea Institute of Oriental Medicine, Daejeon, South Korea; ^3^ College of Pharmacy, Chungnam National University, Daejeon, South Korea; ^4^ Korean Medicine Clinical Trial Center (K-CTC), Korean Medicine Hospital, Kyung Hee University, Seoul, South Korea

**Keywords:** Bojungikki-Tang, colorectal cancer, MC38, PD-L1, immunotherapy, tumor microenvironment

## Abstract

Immune checkpoint blockage targeting PD-L1 has led to breakthroughs in cancer treatment. Although anti-PD-L1-based immunotherapy has been approved as standard therapy in various cancer types, its therapeutic efficacy in most colorectal cancers (CRC) is still limited due to the low response to immunotherapy. Therefore, combining treatment with herbal medicines could be an alternative approach for treating CRC to overcome this limitation. Bojungikki-Tang (BJIKT), a herbal formula used in traditional Chinese medicine, clinically improves the quality of life for cancer patients and has been associated with antitumor and immune-modulating activities. However, the regulatory effect of BJIKT on the immune response in the tumor microenvironment remains largely uninvestigated. In this study, we verified the inhibitory effect of BJIKT on tumor growth and investigated the regulatory effect of combination therapy with BJIKT and anti-PD-L1 on antitumor immune responses in an MC38 CRC-bearing C57BL/6 mouse model. Immune profiling analysis by flow cytometry was used to characterize the exact cell types contributing to anticancer activities. Combination treatment with BJIKT and anti-PD-L1 therapy significantly suppressed tumor growth in MC38-bearing mice and increased the proportion of cytotoxic T lymphocytes and natural killer cells in tumor tissues. Furthermore, BJIKT suppressed the population of myeloid-derived suppressor cells, suggesting that this combination treatment effectively regulates the immunological function of T-cells by improving the tumor microenvironment. The herbal formula BJIKT can be a novel therapeutic option for improving anti-PD-L1-based immunotherapy in patients with CRC.

## Introduction

Immune therapy targeting the immune checkpoint receptor using specific antibodies was a major breakthrough in cancer treatment (Turnis et al., 2015). It is emerging as one of the most powerful approaches for cancer treatment and improving poor outcomes in various cancers (Hegde and Chen, 2020). Immune checkpoint inhibitors (ICIs) that block inhibitory receptors, including programmed death 1 (PD-1), programmed death-ligand-1 (PD-L1), and cytotoxic T-lymphocyte antigen-4, are now available for cancer treatment as a single drug or in combination with other drugs (Robert, 2020). However, unlike other cancer patients, such as non-small cell lung cancer and melanoma, only a small number of colorectal cancer (CRC) patients respond to immunotherapy (Lynch and Murphy, 2016). Many studies have focused on tumor intrinsic factors, including PD-L1 expression, tumor mutational burden, and lack of antigen presentation, but the complexity of CRC makes it difficult to cure patients because tumors exist in a dynamic microenvironment, consisting of tumor cells, immune cells, stroma, and extracellular matrix (Murciano-Goroff et al., 2020). Therefore, a novel therapeutic strategy is urgently needed, and combination regimens may be a promising therapeutic strategy to enhance the immune response of ICIs for CRC treatment (Chen et al., 2021).

Traditional Chinese medicine (TCM) has been clinically used to prevent and treat many diseases for thousands of years. Bojungikki-Tang (BJIKT), which is known as Bu-Zhong-Yi-Qi-Tang in Chinese and Hochu-ekki-to in Japanese, composed of ten herbal medicines, is a widely used TCM to improve the quality of life and immunological status of patients and to strengthen the immune system against infections in China, Japan, and Korea (Kuroiwa et al., 2004; Tatsumi et al., 2009). Recent studies have focused on the potential of BJIKT as a combinatorial treatment and as an adjuvant treatment for cancer-related symptoms in clinical settings (Jeong et al., 2010; Yu et al., 2017; Okabe et al., 2019). Furthermore, other studies have reported that BJIKT augmented the antitumor immune response by enhancing natural killer (NK) cell activity and restoring antitumor T cell response *in vitro* and *in vivo* models (Li et al., 1999; Cho et al., 2004). It also has immune-modulating activities in infectious diseases, lung injury, viral infection, and chronic inflammatory disorders (Yan et al., 2002; Tatsumi et al., 2009; Nakakubo et al., 2020). Although BJIKT could regulate the immune response in cancer, no study has evaluated the combinatorial effect of BJIKT with ICIs. Recent studies support our hypothesis that the active components of BJIKT, such as ginsenosides (Zhang et al., 2019), astrogaloside IV (Liu et al., 2020), atractylenolide III (Liu et al., 2019), and glycyrrhizic acid (Juin et al., 2020) regulate tumor-infiltrating lymphocytes (TILs) and play a crucial role in tumor immunosuppression. It is of great significance to study whether BJIKT combined with ICIs can improve the pharmacological effects of CRC treatment.

Recent research has demonstrated that TILs in CRC and the environment around a tumor, known as the tumor microenvironment, are crucial in CRC development (Ge et al., 2019). The lack of cytotoxic T lymphocytes (CTLs) in the tumor microenvironment suppresses antitumor immunity. Immunosuppressive cells, such as tumor-associated macrophages (TAMs), myeloid-derived suppressor cells (MDSCs), and regulatory T-cells (Tregs), also attenuated the cytotoxicity of CTLs and NK cells in the tumor microenvironment (Yang et al., 2019). Therefore, reprogramming the immunosuppressive tumor microenvironment can enhance the therapeutic effects of immunotherapy, such as ICIs (Phuengkham et al., 2019). In the search for novel therapeutic agents that improve ICI response, herbal medicines have the advantages of multi-targeting potential in the tumor microenvironment and synergistic effects with single-target agents (Zhou et al., 2016). Here, we studied the impact of BJIKT on an MC38 murine syngeneic model and its combination with anti-PD-L1 antibody.

## Materials and Methods

### Chemicals and Reagents

Dulbecco’s modified Eagle’s medium (DMEM), Dulbecco’s phosphate-buffered saline (DPBS), and fetal bovine serum (FBS) were obtained from Gibco (Grand Island, NY, United States). For flow cytometry analysis, BV421 CD45, BV605 CD3, BV710 CD3, PE-Cy7 CD4, AF700 CD8, APC-Cy7 CD8, APC CD335 (NKp46), PerCP-Cy5.5 CD62L, BV510 CD44, FITC Ki-67, PE Granzyme B (GrB), BV650 CD11b, PE-Cy7 F4/80, APC GR1, Zombie NIR Fixable Viability Kit, and TruStain FcX™ PLUS (CD16/32) antibodies were purchased from Biolegend (San Diego, CA, United States). For immunohistochemistry, anti-CD3 and anti-CD8α antibodies were purchased from Abcam (Cambridge, MA, United States). Neutralized antibody anti-mouse PD-L1 (clone 10F.9G2), IgG2b isotype control, and anti-mouse CD8α (Clone 53-6.7) were obtained from BioXCell (West Lebanon, NH, United States). The tumor dissociation kit was obtained from Miltenyi Biotec (Bergisch Gladbach, Germany).

### Bojungikki-Tang Preparation

BJIKT extract was prepared by Hanpoong Pharmaceutical Co., Ltd. (Jeonju, Korea) according to Good Manufacturing Practices. The herbal formula BJIKT is a complex of ten herbal medicines, as listed in Table 1. Astragalus Root (26.6 g), Atractylodes Rhizome White (26.6 g), Ginseng (26.6 g), Angelica Gigas Root (20 g), Bupleurum Root (13.4 g), Jujube (13.4 g), Citrus Unshiu Peel (13.4 g), Licorice (10 g), Cimicifuga Rhizome (6.6 g), and Ginger (3.4 g) were mixed, put into a 10-fold volume of distilled water, and decocted from 80°C to 100°C for 3 h. The solution was filtered through a filter paper (25 μm pore size), evaporated to dryness, and freeze-dried to give a powder (50 g). The yield of BJIKT extract was 31.3%. BJIKT was dissolved in distilled water and DPBS and filtered through a 0.45 μm sterile filter for *in vivo* and *in vitro* studies, respectively. Standard components (purity ≥98.0%) were purchased from Chemfaces (Wuhan, Hubei, China).

**TABLE 1:** Composition of bojungikki-Tang.

Scientific name	Family	English common name	Proportion (%)
*Astragalus membranaceus* (Fisch.) Bunge	Leguminosae	*Astragalus* Root	16.6
*Atractylodes macrocephala* Koidz	Compositae	Atractylodes Rhizome White	16.6
*Panax ginseng* C. A. Mey	Araliaceae	Ginseng	16.6
*Angelica gigas* Nakai	Apiaceae	Angelica Gigas Root	12.5
*Bupleurum falcatum* L	Apiaceae	Bupleurum Root	8.4
*Ziziphus jujuba* var. *inermis* (Bunge) Rehder	Rhamnaceae	Jujube	8.4
*Citrus unshiu* Marcow	Rutaceae	Citrus Unshiu Peel	8.4
*Glycyrrhiza glabra* L	Leguminosae	Licorice	6.3
*Cimicifuga heracleifolia* Kom	Ranunculaceae	Cimicifuga Rhizome	4.1
*Zingiber officinale* Roscoe	Zingiberaceae	Ginger	2.1

### LC-MS/MS Analysis

**FIGURE 1:**
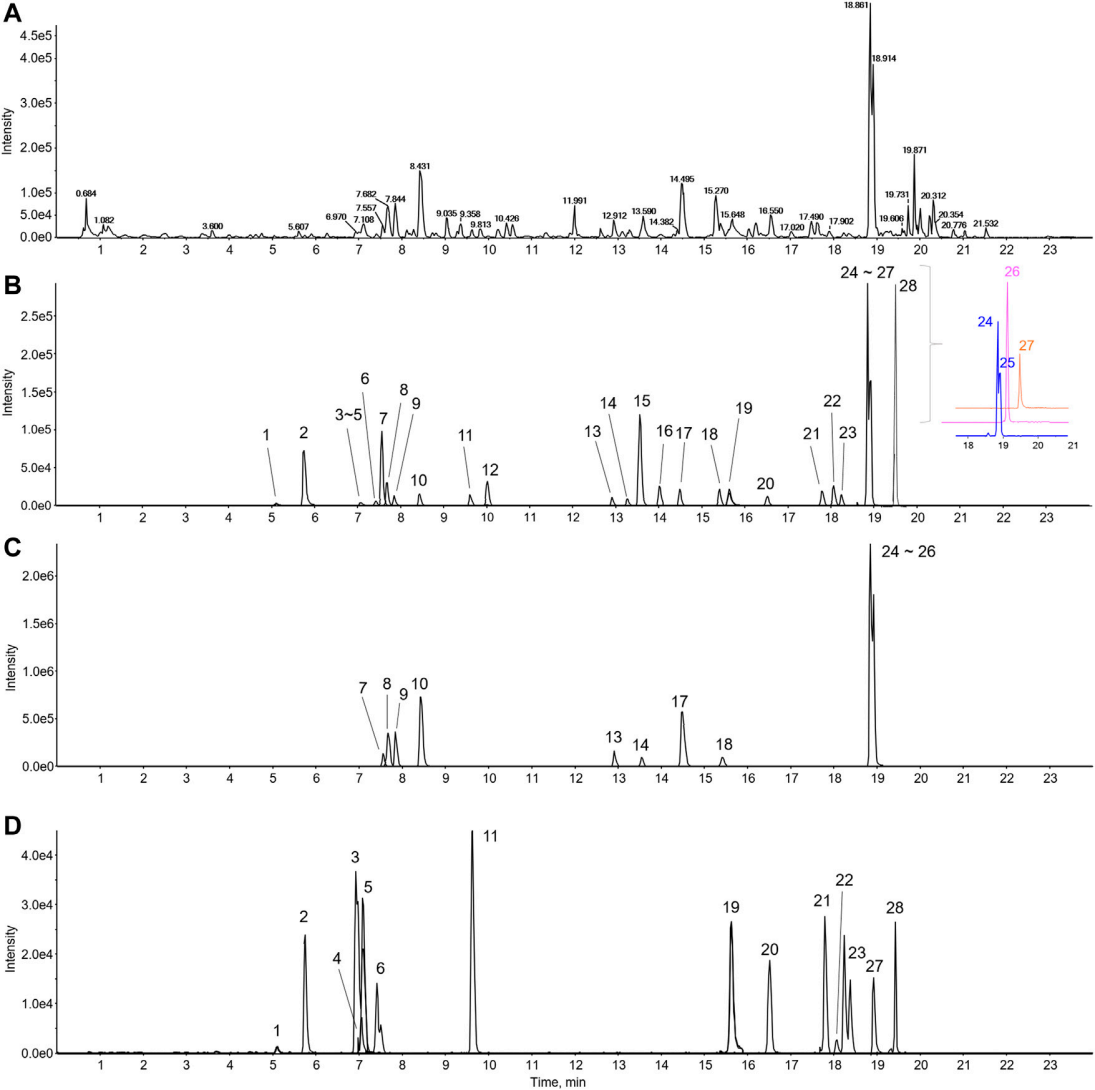
LC-MS/MS chromatogram of BJIKT. The representative base peak chromatogram of the BJIKT extract **(A)** and the extracted ion chromatograms of 28 reference standards **(B)** and sample **(C–D)** were obtained using LC-ESI-MS/MS analysis in positive ion mode. 1: caffeic acid, 2: magnoflorine, 3: ferulic acid, 4: liquiritin apioside, 5: liquiritin, 6: isoferulic acid, 7: cimifugin, 8: nodakenin, 9: narirutin, 10: hesperidin, 11: ginsenoside Rg1, 12: liquiritigenin, 13: ginsenoside Rb1, 14: saikosaponin C, 15: formononetin, 16: astragaloside IV, 17: glycyrrhizin, 18: saikosaponin A, 19: 6-gingerol, 20: atractylenolide III, 21: astragaloside I, 22: saikosaponin D, 23: ginsenoside Rg3, 24: decursin, 25: decursinol angelate, 26: atractylenolide II, 27: 6-shogaol, and 28: atractylenolide I

An ultrahigh-performance liquid chromatography (UHPLC) system (Vanquish, Thermo Fisher Scientific, Sunnyvale, CA, United States) was interfaced with a TripleTOF5600^+^ mass spectrometer (Sciex, Foster City, CA, United States) equipped with a Turbo-V IonSpray. An electrospray ionization (ESI) source in the negative and positive ion mode was utilized to identify major components and quantitation using an information-dependent acquisition (IDA) scan and MRM^HR^ scan concurrently. The parameters were as follows: mass range, 50–1,500 m/z; ion spray voltage, 4.5 kV; source temperature, 450°C; declustering potential, 50 V; nitrogen, nebulizer gas at 50 L/min; heater gas, 50 L/min; curtain gas, 25 L/min; and collision energy, 10 eV. The gradient conditions for chromatographic separation used 0.1% formic acid in water as eluent A and 0.1% formic acid in acetonitrile as eluent B were as follows: 0–1 min; 5% B, 1–4 min; 5%–15% B, 4–11 min; 15%–35% B, 11–17 min; 35%–50% B, 17–19 min; 50%–100% B, 19–24 min; 100% B and equilibration with 5% B for 4 min at a flow rate of 0.4 ml/min. The temperature of the column was 40°C, and the auto-sampler was maintained at 4°C. The injection volume of each sample was 2 μl. The MS and MS/MS data acquisition and processing for qualitative analysis were carried out using Analyst TF 1.7, MasterView, and PeakVeiw 2.2 (SCIEX, Foster City, CA, United States) (Figure 1). The MS/MS data for qualitative analysis were processed to identify and confirm components in the BJIKT extract the retention time, accurate m/z value, isotope distribution, and fragment ions compared to reference standards. We used MultiQuant software for quantitative analysis. The reference standards for each target compound were analyzed, and the amounts of compounds were quantified using corresponding calibration curves of reference standard compounds.

### Cell Culture

MC38 murine CRC cells were obtained from Dr. Eui-Cheol Shin (KAIST, Daejeon, Korea). LLC1 murine lung cancer cells and NK-92 human NK cells were purchased from the American Type Culture Collection (Manassas, VA, United States). HCT116 and KM12SM human CRC cells, CT-26 murine CRC cells, and NCI-H460 human lung cancer cells were purchased from the Korean Cell Bank (Seoul, Korea). MC38 and LLC1 cells were grown in DMEM, and HCT116 cells, KM12SM, CT-26, NCI-H460 cells were grown in RPMI-1640 medium supplemented with 10% FBS, penicillin (100 U/ml), and streptomycin (100 μg/ml) at 37°C in a humidified atmosphere containing 5% CO_2_. NK-92 cells were maintained in Minimum Essential Medium α (Gibco, New York, NY, United States) supplemented with 12.5% horse serum, 12.5% FBS, 0.2 mM myo-inositol, 0.1 mM 2-mercaptoethanol, 0.02 mM folic acid, penicillin 100 U/ml, streptomycin 100 μg/ml, and 100 U/ml recombinant human interleukin 2 (IL-2, Rocky Hill, NJ, United States). NK-92 cells were subcultured every 2 or 3 days, depending on the cell density.

### Animals

Specific pathogen-free four-week-old C57BL/6 mice were purchased from Orient Bio (Seongnam, Korea) and acclimated for one week before experimental use. The mice were housed under the following environmental conditions (temperature, 23°C; humidity, 50%; 12 h light/dark cycle). Animals were fed a standard chow diet (Purina Co., Seoul, Korea) and provided drinking water *ad libitum*. All experimental procedures were approved by the Animal Care and Use Committee of INVIVO (Approval number: IV-RA-12-2004-29) and the Korea Institute of Oriental Medicine (Approval number: 20-042).

### Tumor-Bearing Mice and Treatment

MC38 tumor-bearing mice were prepared by subcutaneous injection of 5 × 10^5^ MC38 cells into the right flank. When the tumor volume reached an average size of 70–85 mm^3^, C57BL/6 mice were evenly divided into four groups and orally administered 0.25, 0.5, and 1.0 g/kg BJIKT daily and/or intraperitoneally injected with 10 mg/kg PD-L1 antibody three times a week. Control mice were administered the same volume of distilled water and 10 mg/kg IgG2b antibody. As for CD8 depletion, 10 mg/kg CD8 antibody was given intraperitoneally 1 day prior to PD-L1 antibody treatment and three times a week. Tumor volume was measured two or three times a week by caliper measurement and calculated using the formula: length × width^2^ × 1/2. The body weight was monitored for toxicity. At the experimental endpoint, tumors were dissected, weighed, and dissociated for analysis. The survival of the mice was recorded daily.

### Cell Proliferation Assay

A cell counting kit-8 assay (CCK-8, Dojindo, Kumamoto, Japan) was used to determine the proliferation of colorectal cancer cells. Briefly, MC38, CT-26, HCT116, and KM12SM cells were treated with BJIKT (125–1,000 μg/ml) for 48 h. Then, 10 µl of CCK-8 reagent was added to each well and incubated for 2 h. The absorbance of the CCK-8 reagent was measured at 450 nm using a Benchmark Plus microplate reader (Bio-Rad, Hercules, CA, United States).

### PD-1/PD-L1 Binding Assay

PD-1/PD-L1 binding activity was determined using the PD-1/PD-L1 Inhibitor Screening Assay Kit (BPS Bioscience, San Diego, CA, United States) according to the manufacturer’s protocol. Briefly, recombinant hPD-L1 (100 ng/well) was coated onto a 96-well plate by overnight incubation at 4°C. After washing and blocking for 1 h at room temperature, BJIKT (0–2,000 μg/ml) or atezolizumab (0–2,000 ng/ml, Tecentriq^®^, Roche, Basel, Switzerland) was added and reacted for 1 h. Atezolizumab was used as a positive control. Then, biotinylated hPD-1 (10 ng/well) was incubated with PD-L1 for 2 h on a plate. Finally, the plate was treated with streptavidin-horseradish peroxidase (HRP), followed by the addition of ELISA ECL substrate to produce chemiluminescence. The relative chemiluminescence was measured using a SpectraMax i3 plate reader (Molecular Devices, Sunnyvale, CA, United States).

### Preparation of Leucocytes

Single-cell suspensions of leucocytes from tumor tissue, tumor-draining lymph nodes (TDLNs), and spleens were prepared. Briefly, tumors were cut into small pieces and digested with a tumor dissociation kit (Miltenyi Biotec) to prepare single cells from tumor tissues. Then, the tumor tissues were gently passed through a sterile 70 µm strainer. Cells were collected by centrifugation at 350 ×g for 4 min at 4°C, and red blood cells (RBCs) were removed using RBC lysis buffer (BioLegend, San Diego, CA, United States). The cells were washed twice with DPBS and resuspended in FACS buffer (2% FBS, 0.5 mM EDTA in DPBS) for flow cytometry analysis. TDLNs and spleens were removed and ground gently by passing a sterile 70 µm strainer. The cells were collected by centrifugation at 350 ×g for 4 min at 4°C, and RBCs of spleens were removed. For flow cytometry analysis, the cells were then washed twice with DPBS and resuspended in FACS buffer.

### Flow Cytometry Analysis

Immune population analysis was performed by flow cytometry. The samples were blocked by non-specific staining with TruStain FcX™ PLUS and incubated for 30 min at 4°C in the dark with brilliant violet staining buffer (Biolegend) containing fluorescence-conjugated antibodies (1:100) against the surface markers CD45, CD3, CD4, CD8, NKp46, CD62L, CD44, CD11b, F4/80, and GR-1. The samples stained for intracellular GrB and Ki-67 expression were permeabilized with eBioscience™ Foxp3/Transcription Factor Staining Buffer Set (eBioscience, San Diego, CA, United States). Finally, the cells were detected using a BD LSRFortessa™ X-20 flow cytometer (BD Biosciences, San Diego, CA, United States).

### Immunohistochemistry

The tumor tissues were fixed with 10% neutral buffered formalin (Sigma-Aldrich) for 24 h, and paraffin-embedded sections were prepared. Immunohistochemical staining of lymphocytes in tumor tissues was performed by incubating the sections with anti-CD3 and anti-CD8α antibodies. The sections were visualized using a DAB substrate kit (Vector Laboratories, Burlingame, CA, United States).

### NK Cell-Mediated Cytotoxicity

NK cell-mediated cytotoxicity was evaluated using a calcein-AM (Invitrogen, Carlsbad, CA) release assay. Briefly, NK-92 effector cells were cocultured with calcein-AM-labeled NCI-H460 target-cells at effector/target (E:T) ratio of 5:1 for 4 h at 37°C. Calcein fluorescence signal from lysed target-cells was measured using a SpectraMax i3 plate reader (excitation: 485 nm; emission: 530 nm). NK cell cytotoxicity was calculated as follows: NK cell cytotoxicity (%) = (measured value – spontaneous value)/(maximum value – spontaneous value) × 100. Spontaneous value was measured from the calcein released from only calcein-labeled target-cells in complete growth medium. To measure maximum value, calcein-labeled target-cells were incubated in complete growth medium containing 2% Triton X-100 (Sigma-Aldrich).

### Cytokine Level

The serum was collected from MC38-bearing mice. Cytokine levels (IL-1β, IL-2, IL-4, IL-6, IL-17A, IFN-γ, and TNF-α) were determined using Bio-Plex Pro Mouse Cytokine Assay kit according to manufacturer’s instructions (Bio-Rad). The measurements were performed on a Bio-Plex 200 system (Bio-Rad). The expression levels of TGF-β and IL-10 in the lysate of tumor tissues were measured by ELISA kit (Abcam).

### Magnetic Isolation and Culture of Myeloid-Derived Suppressor Cells

MDSCs were purified from the spleens of MC38-implanted mice when the average tumor volume reached approximately 800 mm^3^. Briefly, MDSCs from splenocytes were separated using the EasySep™ Mouse MDSC Isolation Kit (STEMCELL Technologies, Vancouver, Canada), and the sorted MDSCs were confirmed by flow cytometry analysis with anti-CD11b and anti-GR-1 antibodies. MDSCs were incubated in RPMI-1640 medium containing 10% FBS and 40 ng/ml recombinant mouse granulocyte-macrophage colony-stimulating factor (GM-CSF, PeproTech).

### Quantitative PCR

Total RNA was extracted from tumor tissue using the RNeasy Plus Mini Kit (Qiagen, Valencia, CA, United States). Thirty milligrams of tumor tissue was homogenized, and the RNA was prepared using QIAzol reagent according to the manufacturer’s instructions. The purity and RNA concentration were measured using Nanodrop (Thermo Scientific, Waltham, MA, United States), and 1 μg of RNA was converted to cDNA using the iScript™ Advanced cDNA Synthesis kit (Bio-Rad). The sequences of the primers were as follows: ARG-1, forward 5′-CAA GAC AGG GCT CCT TTC AG-3′, reverse 5′-TGG CTT ATG GTT ACC CTC CC-3′; NOS2, forward 5′- GAT GTT GAA CTA TGT CCT ATC TCC-3′, reverse 5′-GAA CAC CAC TTT CAC CAA GAC-3′; ACTB, forward 5′-CCT TCT TGG GTA TGG AAT CCT G-3′, reverse 5′-CAA TGC CTG GGT ACA TGG TG-3′. Gene expression analysis was performed using SsoAdvanced SYBR Green Supermix kit (Bio-Rad) on a Bio-Rad CFX Connect Real-Time System (Bio-Rad) under the following reaction conditions: initial denaturation and enzyme activation at 95°C for 2 min, followed by 40 cycles of amplification at 95°C for 5 S and 60°C for 30 S. Gene expression levels were calculated using 2^−ΔΔCt^ method and normalized by the housekeeping gene ACTB as follows: ΔCt = Ct value of target gene − Ct value of ACTB; ΔΔCt = ΔCt of control group − ΔCt of BJIKT group.

### Systematic Pharmacological Analysis of Bojungikki-Tang

The target genes of twenty-eight compounds of BJIKT were selected through target fishing methods by integrating two herbal databases: the Encyclopedia of Traditional Chinese Medicine (ETCM) (Xu et al., 2019) and Symptom Mapping (SymMap) (Wu et al., 2019). A total of 711 CRC-related targets were collected in GeneCards using the filtering criteria of “relevance score ≥18”. Venny 2.1.0 (http://bioinfogp.cnb.csic.es/tools/venny/) was used to draw a Venn diagram. Cytoscape software was used to construct a network to connect 10 herbs and 28 compounds of BJIKT with the selected 246 target genes. Pathway enrichment analysis was performed using *the enrichR* package in R with the gene sets of the Kyoto Encyclopedia of Genes and Genomes (KEGG) and Gene Ontology Biological Process (GO BP) (Kuleshov et al., 2016). The distribution of the common target genes between BJIKT and CRC was analyzed using the *pathview* package in R (Luo & Brouwer, 2013).

### Statistical Analysis

Statistical analyses were performed using GraphPad Prism 8. Data with error bars represent mean ± standard deviation (SD), except for tumor volume curves, which represent the mean ± standard error of the mean (SEM). Statistical analysis of significance was based on a two-tailed Student’s *t-test*. Animal survival was presented using Kaplan–Meier survival curves and analyzed by the Gehan–Breslow–Wilcoxon test. A value of *p* < 0.05, was chosen as the criterion for statistical significance.

## Results

### LC-MS/MS Analysis of Bojungikki-Tang

We determined the twenty-eight major compounds, including astragaloside I, astragaloside IV, formononetin, atractylenolide I, atractylenolide II, atractylenolide III, ginsenosides Rb1, ginsenoside Rg1, ginsenoside Rg3, nodakenin, decursin, decursinol angelate, saikosaponin A, saikosaponin C, saikosaponin D, magnoflorine, hesperidin, narirutin, liquiritin, liquiritigenin, glycyrrhizin, liquiritin apioside, cimifugin, ferulic acid, isoferulic acid, caffeic acid, 6-gingerol, and 6-shogaol. The chemical information and content quantified by LC-MS/MS analysis are listed in Table 2. The representative components were hesperidin (*Citrus unshiu* Marcow.), glycyrrhizin (*Glycyrrhiza glabra* L.), and narirutin (*Citrus unshiu* Marcow.), and nodakenin, decursin, and decursinol angelate (*Angelica gigas* Nakai).

**TABLE 2:** Qualitative and quantitative analysis of chemical components authentically in BJIKT.

Peak no.	Compound Name	Molecular Formula	Rt (min)	Adduct	Measured Molecular ions (m/z)	Error (ppm)	Fragment ion Used in Mrm Mode (m/z)	Linear Range (ng/ml)	Regression Equation	*R* ^*2*^	Content (mg/g)	Origin
1	caffeic acid	C_9_H_8_O_4_	5.10	[M + H]^+^	181.0487	−2.6	163.0390	50–7,500	y = 3.26x+298.04	0.9966	0.0273	Cimicifuga Rhizome
2	magnoflorine	C_20_H_24_NO_4_	5.74	[M]^+^	342.1700	−1.1	342.1698	10–500	y = 180.03x+1901.15	0.9974	0.0105	Jujube
3	ferulic acid	C_10_H_10_O_4_	7.07	[M + H]^+^	195.0650	−1.3	177.0558	50–2,500	y = 5.65x+131.90	0.9947	0.1268	Cimicifuga Rhizome
4	liquiritin apioside	C_26_H_30_O_13_	7.10	[M + H]^+^	551.1754	−1.3	257.0809	50–1,000	y = 2.82x+102.51	0.9973	1.8770	Licorice
5	liquiritin	C_21_H_22_O_9_	7.14	[M + H]^+^	419.1336	−0.8	257.0813	50–5,000	y = 16.60x+858.41	0.9993	0.4866	Licorice
6	isoferulic acid	C_10_H_10_O_4_	7.41	[M + H]^+^	195.0650	−1.4	177.0558	70–1,000	y = 7.64x+803.10	0.9956	0.1504	Cimicifuga Rhizome
7	cimifugin	C_16_H_18_O_6_	7.56	[M + H]^+^	307.1179	0.8	307.1179	25–2,500	y = 212.77x+4,574.75	0.9986	0.0669	Cimicifuga Rhizome
8	nodakenin	C_20_H_24_O_9_	7.68	[M + H]^+^	409.1491	0.5	247.0976	75–10,000	y = 31.22x+2,897.68	0.9982	1.9347	Angelica Gigas Root
9	narirutin	C_27_H_32_O_14_	7.84	[M + H]^+^	581.1862	−0.5	273.0756	100–20,000	y = 4.44x+370.52	0.9983	2.6557	Citrus Unshiu Peel
10	hesperidin	C_28_H_34_O_15_	8.43	[M + H]^+^	611.1969	0.0	303.0867	100–20,000	y = 5.75x+500.37	0.9990	7.2197	Citrus Unshiu Peel
11	ginsenoside Rg1	C_42_H_72_O_14_	9.62	[M + Na]^+^	823.4820	0.2	823.4784	250–7,500	y = 4.44x+370.52	0.9964	0.3464	Ginseng
12	liquiritigenin	C_15_H_12_O_4_	10.01	[M + H]^+^	257.0808	0.8	257.0819	250–5,000	y = 33.44x+6,515.63	0.9965	0.0264	Licorice
13	ginsenoside Rb1	C_54_H_92_O_23_	12.91	[M + Na]^+^	1,131.5958	0.9	1,131.5919	50–200,000	y = 5.67x-1211.30	0.9960	0.7744	Ginseng
14	saikosaponin C	C_48_H_78_O_17_	13.27	[M + Na]^+^	949.5149	0.5	949.5110	75–200,000	y = 5.91x-121.57	0.9981	0.1391	Bupleurum Root
15	formononetin	C_16_H_12_O_4_	13.55	[M + H]^+^	269.0809	0.7	269.0819	25–1,000	y = 383.08x+10,316.81	0.9971	0.0349	*Astragalus* Root
16	astragaloside IV	C_41_H_68_O_14_	14.02	[M + Na]^+^	807.4516	−0.1	807.4491	500–5,000	y = 14.77x+7,692.23	0.9973	0.0180	*Astragalus* Root
17	glycyrrhizin	C_42_H_62_O_16_	14.49	[M + H]^+^	823.4128	−1.0	453.3364	75–20,000	y = 9.59x+900.11	0.9995	5.6550	Licorice
18	saikosaponin A	C_42_H_68_O_13_	15.42	[M + Na]^+^	803.4562	−1.2	803.4505	75–10,000	y = 13.67x+1,235.43	0.9989	0.5603	Bupleurum Root
19	6-gingerol	C_17_H_26_O_4_	15.62	[M + Na]^+^	317.1724	0.1	317.1709	100–1,000	y = 19.48x+2,539.63	0.9978	0.1073	Ginger
20	atractylenolide III	C_15_H_20_O_3_	16.51	[M + H]^+^	249.1485	−1.0	231.1387	75–7,500	y = 8.58x+415.28	0.9983	0.0934	Atractylodes Rhizome White
21	astragaloside I	C_45_H_72_O_16_	17.81	[M + Na]^+^	891.4733	−0.4	891.4697	100–10,000	y = 15.18x+4,344.61	0.9987	0.0605	*Astragalus* Root
22	saikosaponin D	C_42_H_68_O_13_	18.06	[M + Na]^+^	803.4552	−1.9	803.4545	100–2,500	y = 16.84x+834.08	0.9976	0.0044	Bupleurum Root
23	ginsenoside Rg3	C_42_H_72_O_13_	18.24	[M + Na]^+^	807.4868	−0.8	807.4854	500–5,000	y = 9.50x+2084.09	0.9970	0.1029	Ginseng
24	decursin	C_19_H_20_O_5_	18.87	[M + H]^+^	329.1390	−0.4	229.0860	500–2,500	y = 330.01x+110,860	0.9979	1.7510	Angelica Gigas Root
25	decursinol angelate	C_19_H_20_O_5_	18.91	[M + H]^+^	329.1390	−0.4	229.0860					
26	atractylenolide II	C_15_H_20_O_2_	18.88	[M + H]^+^	233.1536	−1.6	187.1476	10–500	y = 90.31x+244.87	0.9990	0.0029	Atractylodes Rhizome White
27	6-shogaol	C_17_H_24_O_3_	19.01	[M + H]^+^	277.1796	−0.7	277.1796	50–1,000	y = 3.42x+109.73	0.9983	0.0061	Ginger
28	atractylenolide I	C_15_H_18_O_2_	19.42	[M + H]^+^	231.1376	−0.6	231.1379	10–500	y = 557.24x+3,935.91	0.9979	0.0020	Atractylodes Rhizome White

### Bojungikki-Tang Affected the Tumor Growth of Subcutaneous MC38 Cancer Cells

**FIGURE 2:**
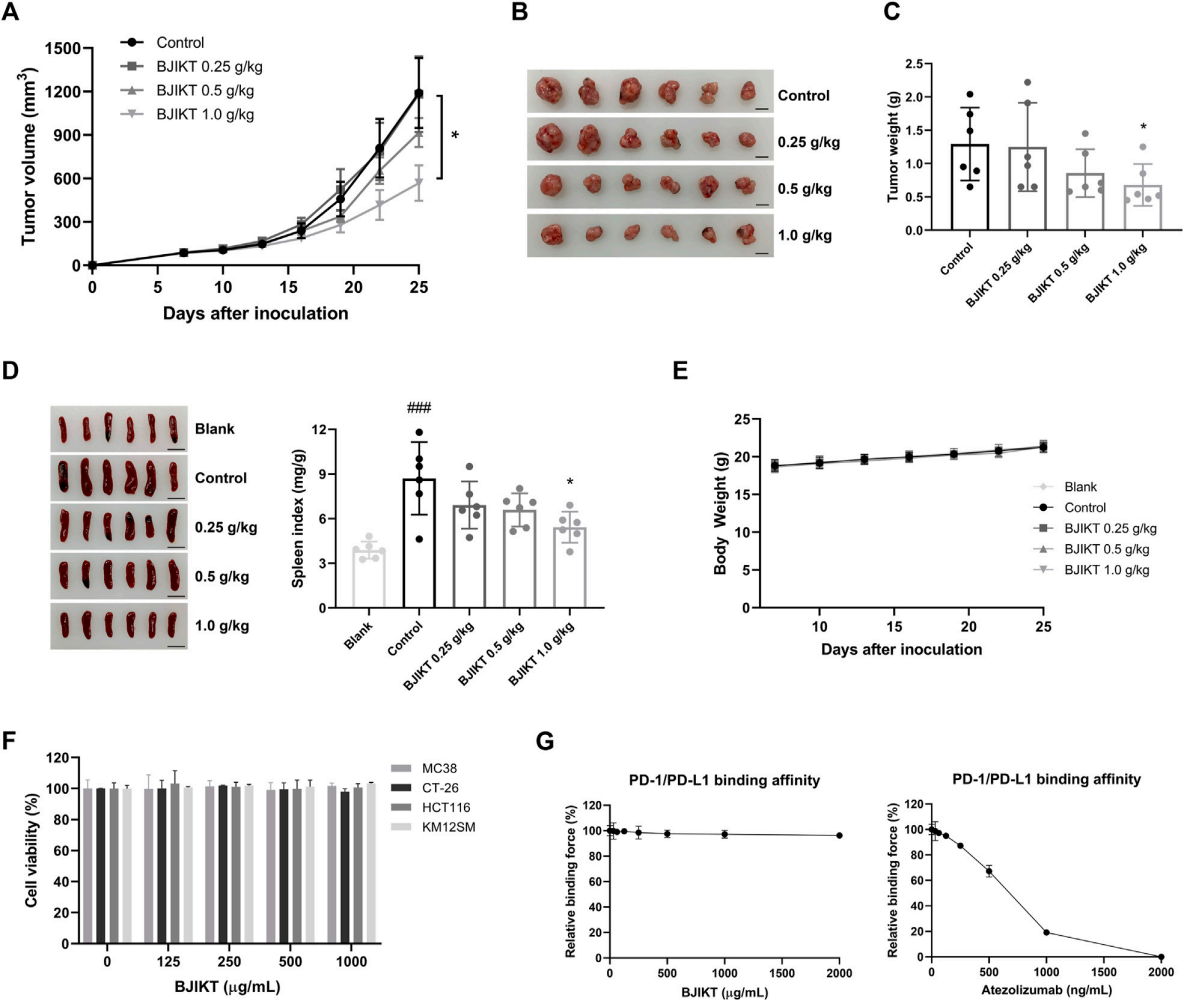
BJIKT inhibited the tumor growth of the MC38 colorectal tumor. MC38 cells (5 × 10^5^ cells) were inoculated subcutaneously into the right flank of C57BL/6 mice. Mice were divided into the control and BJIKT-treated groups (*n* = 6, each group). One week after injection (a tumor volume nearly of 85 mm^3^), BJIKT (0.25, 0.5, and 1.0 g/kg per day) were daily administered, and distilled water was used as a vehicle control. **(A)** Tumor volumes were measured every 3 days after the treatment. **(B)** Tumor images from mice. Scale bar represents 10 mm. **(C)** Tumor weights were shown. **(D)** Picture of the spleen and spleen index of mice were shown. Scale bar represents 10 mm. **(E)** Body weight changes of MC38-bearing mice. **(F)** Effect of BJIKT on the proliferation of colorectal cancer MC38, CT-26, HCT116, and KM12SM cells. CCK-8 was used to detect cell viability. **(G)** Effect of BJIKT on PD-1/PD-L1 binding affinity. The indicated concentrations of BJIKT were treated on plates coated with PD-L1 and then reacted with PD-1. Atezolizumab was used as a positive control. ^###^ *p* < 0.001, compared to the blank group; *p < 0.05, compared to the control group.

To investigate the inhibitory effect of BJIKT on tumor growth, we confirmed tumor growth in MC38-bearing mice. After 18 days of administration of BJIKT, tumor growth was significantly suppressed in 1.0 g/kg BJIKT-treated group (Figures 2A,B). BJIKT also decreased the tumor weight compared to that in the control group (Figure 2C). Additionally, MC38 tumor-bearing mice had a significantly increased spleen index, whereas 1.0 g/kg BJIKT treatment reduced the spleen index, suggesting that its antitumor effect was related to the regulation of immunologic function (Figure 2D). No significant decrease was observed in the body weight of each group (Figure 2E). We investigated whether BJIKT directly affected CRC cell proliferation. The results showed that the proliferation of MC38 and CT-26 murine CRC cells and HCT116 and KM12SM human CRC cells was unaffected by BJIKT (Figure 2F). Moreover, BJIKT did not affect the PD-1/PD-L1 binding affinity (Figure 2G), suggesting that BJIKT exerts antitumor effects by regulating the immune response in the tumor microenvironment, rather than exerting direct cytotoxic effects on CRC cells.

### Bojungikki-Tang Combined With Anti-PD-L1 Antibody Dramatically Inhibited the MC38 Tumor Growth in Mice.

**FIGURE 3:**
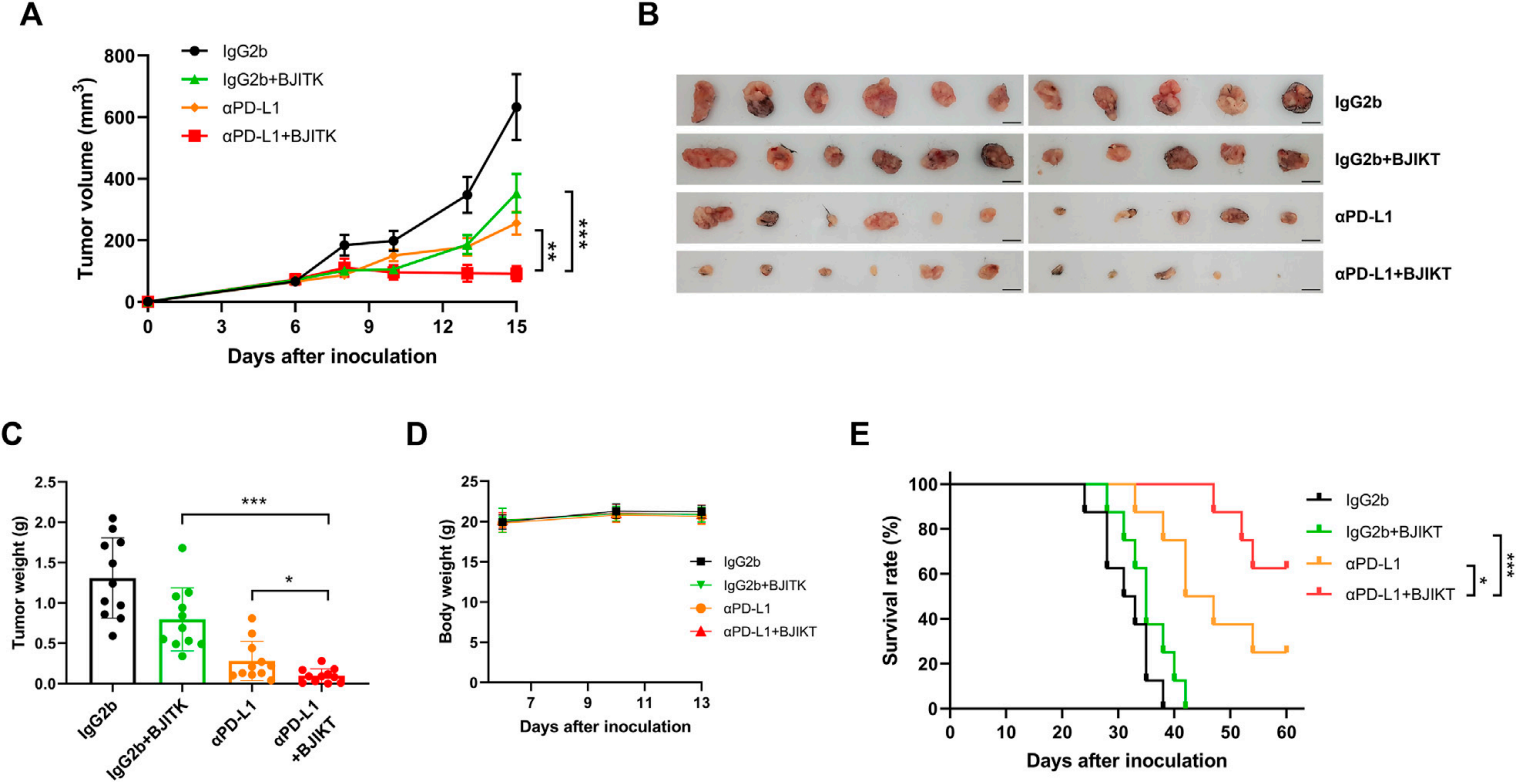
BJIKT combined with anti-PD-L1 (αPD-L1) antibody dramatically suppressed the tumor growth in the immunocompetent MC38-bearing mice. MC38 cells (5 × 10^5^ cells) were injected subcutaneously into C57BL/6 mice. Six days after injection, mice were divided into four groups (*n* = 11, each group) as follows: control mice, mice with BJIKT, mice with PD-L1 antibody, and mice with combination treatment (BJIKT and PD-L1 antibody). BJIKT (1.0 g/kg) was orally administered daily and anti-PD-L1 antibody (10 mg/kg) was intraperitoneally injected 3 times a week. **(A)** Tumor volumes were measured two or three times a week after the treatment in MC38-bearing mice. **(B)** Tumor images from mice. Scale bar represents 10 mm. **(C)** Tumor weights were shown. **(D)** Body weight changes of MC38-bearing mice. **(E)** The Kaplan–Meier survival distribution in the model is displayed for the mice following the treatment (*n* = 8, each group). *p < 0.05, **p < 0.01, and ***p < 0.001, compared to the combination group.

To further investigate the effect of BJIKT in the tumor immune system, we built two subcutaneous syngeneic models in immunocompetent MC38-bearing mice and poorly immunogenic LLC1-bearing mice and investigated the combinatorial effect of 1.0 g/kg BJIKT with anti-PD-L1 antibody on tumor growth and weight *in vivo*. The mice were divided into four groups to which IgG2b was administered as a control, IgG2b with BJIKT, anti-PD-L1, and anti-PD-L1 with BJIKT. After 9 days of administration, the group that received 1.0 g/kg BJIKT or anti-PD-L1 antibody alone showed an inhibitory effect on MC38 tumor growth. The inhibitory effect was significantly enhanced when BJIKT or anti-PD-L1 antibodies were combined (Figures 3A,B). However, in LLC1-bearing mice, BJIKT and/or anti-PD-L1 had no inhibitory effects on tumor growth (Supplementary Figure S1A), suggesting that BJIKT-induced tumor regression and its combinatorial effect are immune-mediated. Tumor weight was also measured after the mice were sacrificed. MC38 tumor weight was further decreased in the combination group (Figure 3C), whereas LLC1 tumor weight was not (Supplementary Figure S1B). No significant decrease was observed in the body weight of each group during administration (Figure 3D; Supplementary Figure S1C). Additionally, an independent study was performed to determine the impact of combination therapy on the survival. We observed that the combination therapy was significantly more effective than BJIKT or anti-PD-L1 antibody monotherapy and prolonged overall survival (Figure 3E). These findings suggest that combination therapy provides more beneficial effects over monotherapy.

### Bojungikki-Tang Promoted the Infiltration of CD3^+^ and CD8^+^ T-cells With Anti-PD-L1 Antibody

**FIGURE 4:**
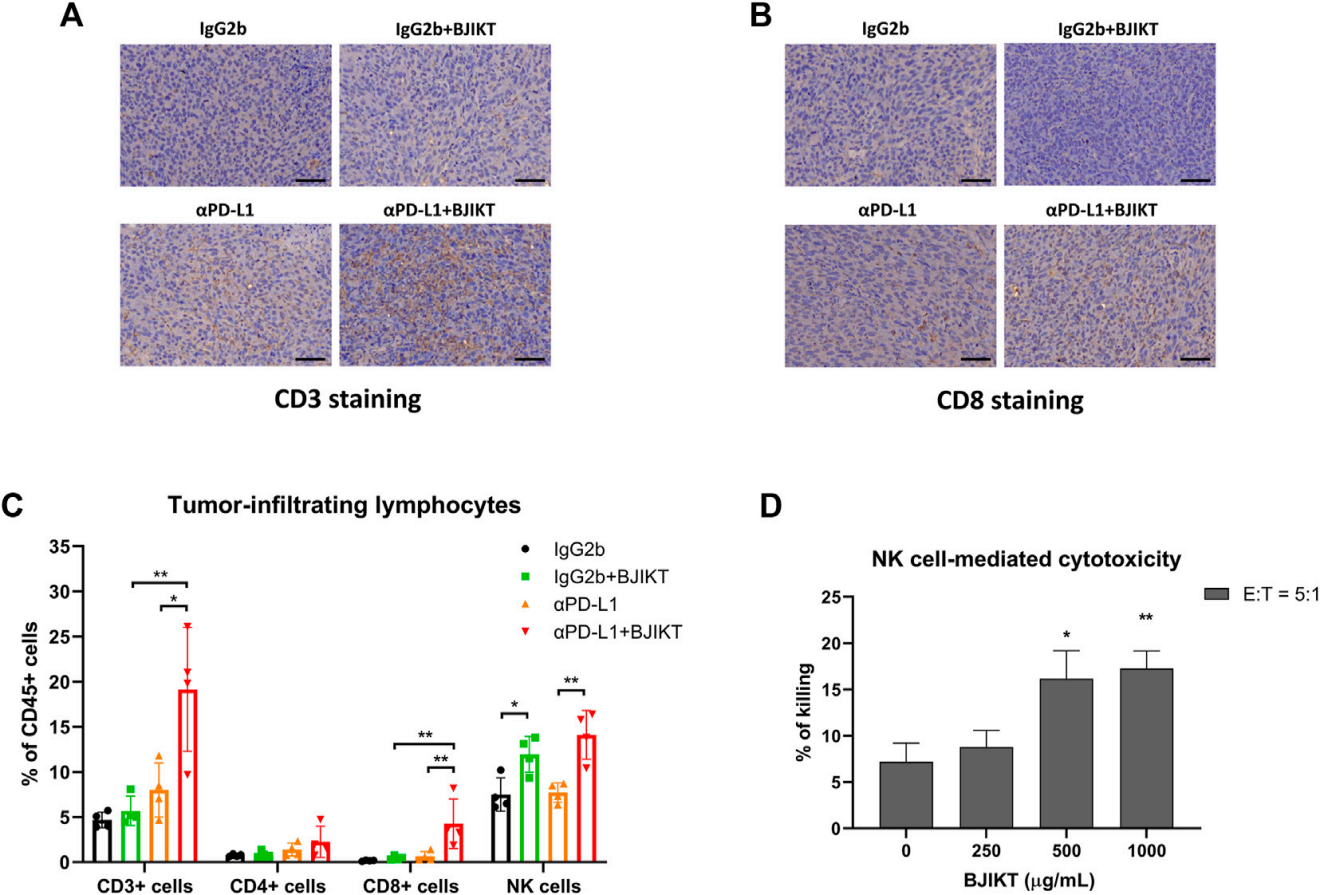
BJIKT combined with anti-PD-L1 antibody prompted the antitumor immune response by regulating tumor-infiltrating lymphocytes in tumor tissue. **(A,B)** Tumor tissues were fixed and immunohistochemical staining was performed. Representative images of CD3^+^ T-cells **(A)** and CD8^+^ T-cells **(B)** immunostaining in MC38 tumor tissues. Scale bar represents 50 μm. **(C)** The infiltration of tumor-associated lymphocytes, including T-cells and NK cells in tumor tissues after BJIKT and/or anti-PD-L1 treatment, was confirmed using flow cytometry. *p < 0.05 and **p < 0.01, compared between each group. **(D)** Killing activity of NK cells to NCI-H460 human lung cancer cells by BJIKT. NK-92 cells were cocultured with calcein-AM-labeled NCI-H460 cells at effector/target (E:T) ratio of 5:1 for 4 h at 37°C. *p < 0.05 and **p < 0.01, compared to the control group.

We examined the tumor infiltration of CD3^+^ and CD8^+^ T-cells to investigate the antitumor immune response of BJIKT combined with anti-PD-L1 antibody *in vivo*. The results showed that BJIKT monotherapy did not change the infiltration of CD3^+^ and CD8^+^ T-cells, but anti-PD-L1 monotherapy slightly increased the infiltration of them in IHC staining. However, the combination of BJIKT and anti-PD-L1 antibodies resulted in a more substantial increase in the infiltration of CD3^+^ T-cells (Figure 4A) and CD8^+^ T-cells (Figure 4B; Supplementary Figure S2). These results suggest that BJIKT regulates the tumor microenvironment, which is closely associated with T cell responses, thereby enhancing the pharmacological effects of anti-PD-L1 antibodies.

### Bojungikki-Tang Combined With Anti-PD-L1 Antibody Promoted Antitumor Immune Response by Regulating Tumor-Infiltrating Lymphocytes

To investigate the immune-modulating effect in the tumor microenvironment after BJIKT and/or anti-PD-L1 treatment, we analyzed the proportion of tumor-associated lymphocytes, such as T-cells and NK cells in tumor tissues. As shown in Figure 4C, quantitative analysis also indicated that BJIKT with anti-PD-L1 promoted an antitumor immune response by increasing the population of CD3^+^ T-cells (CD3^+^NKp46^−^) and CD8^+^ T-cells (CD3^+^NKp46^−^CD4^−^CD8^+^). Although CD3^+^ and CD8^+^ T-cells were increased in the αPD-L1 group, compared to the BJIKT group, there was no significant difference. However, the population of CD4^+^ T-cells (CD3^+^NKp46^−^CD4^+^CD8^−^) did not change. Interestingly, the BJIKT alone group showed an increased NK cell (CD3^−^NKp46^+^) population, whereas combination treatment with anti-PD-L1 did not increase further. To confirm the effect of BJIKT on the activity of NK cells, we used a well-established experimental method using NK-92 and H460 cells. Our results showed that BJIKT promoted the killing activity of NK-92 cells against NCI-H460 cancer cells (Figure 4D). We also performed an immunoassay of TILs in LLC1-bearing mice. However, only a small proportion of TILs was detected in the poorly immunogenic LLC1-bearing mice (Supplementary Figure S4A). These results suggest that the antitumor effect of BJIKT could be associated with the regulation of T lymphocytes and NK cells.

### Bojungikki-Tang Regulated the Immune Responses of CD8^+^ T-cells With Anti-PD-L1 Antibody.

**FIGURE 5:**
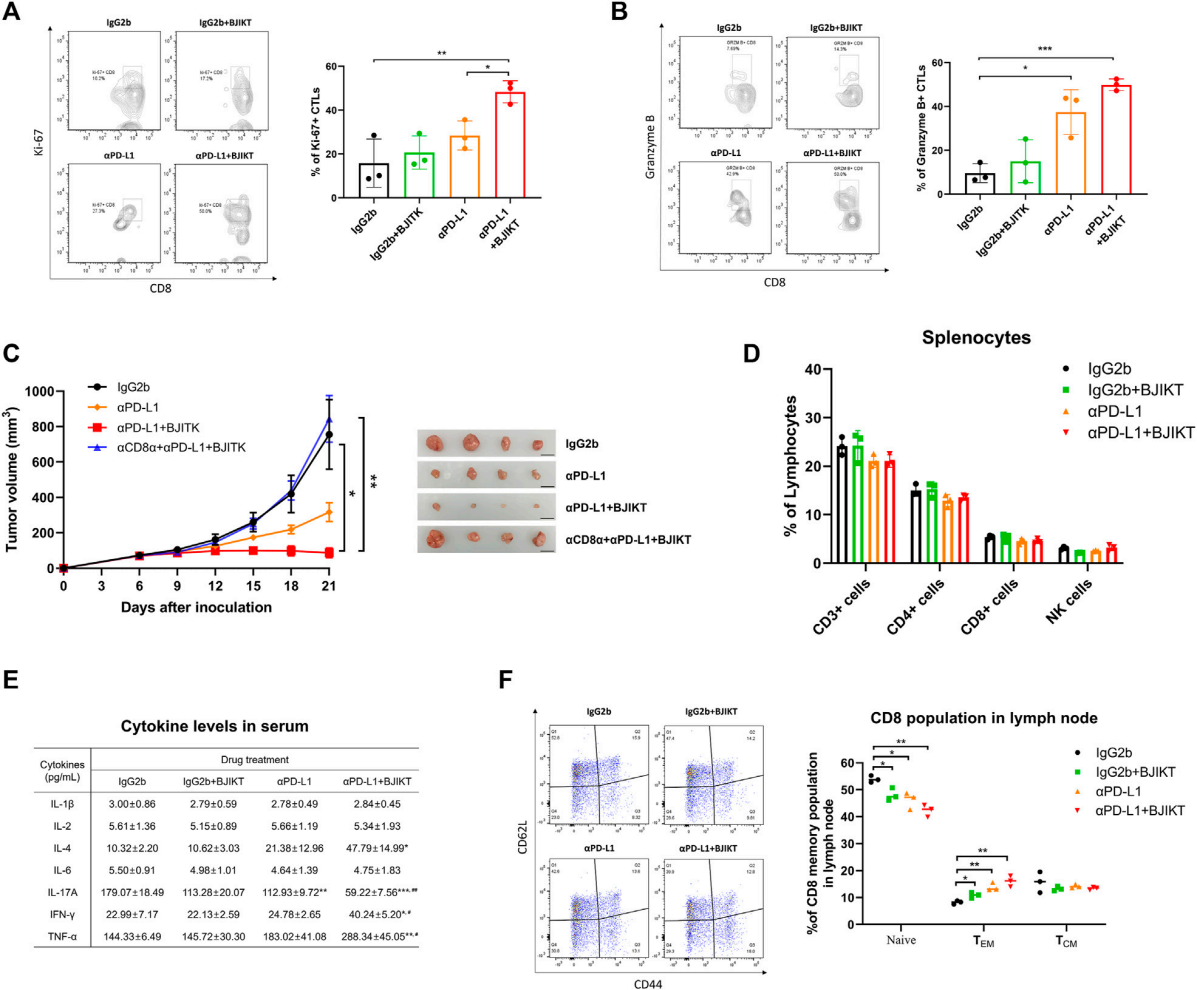
BJIKT combined with anti-PD-L1 regulated the immune responses of CD8^+^ T-cells with anti-PD-L1 in MC38 tumor-bearing mice. **(A,B)** Flow cytometric analysis of CD8^+^ T-cells in tumor tissue after the treatment. Ki-67-expressing **(A)** and granzyme B-expressing **(B)** CD8^+^ T-cells. **(C)** Effect of CD8 depletion prior to combination treatment. Anti-CD8α antibody (10 mg/kg) was given intraperitoneally 1 day prior to PD-L1 antibody treatment and three times a week. **(D)** Effect of BJIKT on the systemic immune response in the splenocytes of MC38-bearing mice. **(E)** Change of inflammatory cytokines in serum collected from MC38-bearing mice. Cytokine levels (IL-1β, IL-2, IL-4, IL-6, IL-17A, IFN-γ, and TNF-α) were determined using Bio-Plex Pro Mouse Cytokine Assay kit. *p < 0.05, **p < 0.01, and ***p < 0.001, compared to the IgG2b group; ^#^ *p* < 0.05 and ^##^ *p* < 0.01, compared to the αPD-L1 group. **(F)** The frequency of memory CD8^+^ T-cells in the tumor-draining lymph node. *p < 0.05 and **p < 0.01, compared between each group.

To assess the activation of CD8^+^ T-cells after anti-PD-L1-based immunotherapy, we analyzed the CTLs in tumor tissue and checked intracellular expression of Ki-67, which is a cell proliferation marker expressed by recently divided cells for cell proliferation, and GrB, which is released by activated CTLs and NK cells to kill target-cells. While anti-PD-L1 antibody treatment increased the number of Ki-67-expressing CD8^+^ T-cells, the combination of BJIKT and anti-PD-L1 was even more pronounced (Figure 5A), suggesting that BJIKT enhanced the proliferation of CD8^+^ T-cells with anti-PD-L1 antibodies. Additionally, we observed that anti-PD-L1 significantly increased GrB secretion by CTLs, and this combination further improved its performance (Figure 5B). These results suggest that combination treatment with BJIKT enhanced CTL-dependent anti-tumor immune response by regulating CTLs activation. This phenomenon was correlated with the inhibition of MC38 tumor growth, indicating that the combination therapy not only increased the number of CTLs, but improved their activities. Next, we investigated the importance of CD8^+^ T-cells mediating the combinatorial effect of BJIKT with anti-PD-L1 antibody. *In vivo* CD8 depletion experiment, we observed that the depletion of CD8^+^ T-cells abolished the antitumor effect of combination treatment (Figure 5C). To further understand the effect of BJIKT on the immune responses of T-cells in MC38 tumor-bearing mice, we evaluated how BJIKT treatment systemically affected the composition of T-cells in the spleen of MC38-bearing mice. In splenic lymphocytes, we observed no changes in the subsets of lymphocytes between the treatment groups (Figure 5D). In addition, we measured the well-known immunomodulatory cytokines in serum. The results showed that the combination treatment of BJIKT and anti-PD-L1 antibody significantly increased the levels of IL-4, IFN-γ, and TNF-α than all other treatments. However, the level of IL-17A was significantly decreased. IL-1β, IL-2, and IL-6 were not significantly changed in the serum (Figure 5E). CD8^+^ T-cells can be further categorized into memory and naïve phenotypes based on CD44 and CD62L with CD44^low^CD62L^high^ population considered naïve, CD44^high^CD62L^high^ population considered T central memory cells (T_CM_), and CD44^high^CD62L^low^ population considered T effector memory cells (T_EM_). In TDLN of MC38-bearing mice, the frequency of memory CD8^+^ T-cells was also assessed to confirm the long-lasting immune response after the combination treatment. As shown in Figure 5F, we observed a significant increase in the percentage of CD8^+^ T_EM_ (CD44^high^CD62L^low^) with a corresponding decrease in the proportion of naïve CD8^+^ T-cells (CD44^low^CD62L^high^) in BJIKT treatment and/or anti-PD-L1 antibody, but not in CD8^+^ T_CM_ (CD44^high^CD62L^high^). These results suggest that combination treatment of BJIKT and anti-PD-L1 antibody could promote the effector function of CD8^+^ T cell memory phenotype in lymph nodes.

### Bojungikki-Tang Attenuated the Myeloid-Derived Suppressor Cells in the Tumor Microenvironment

**FIGURE 6:**
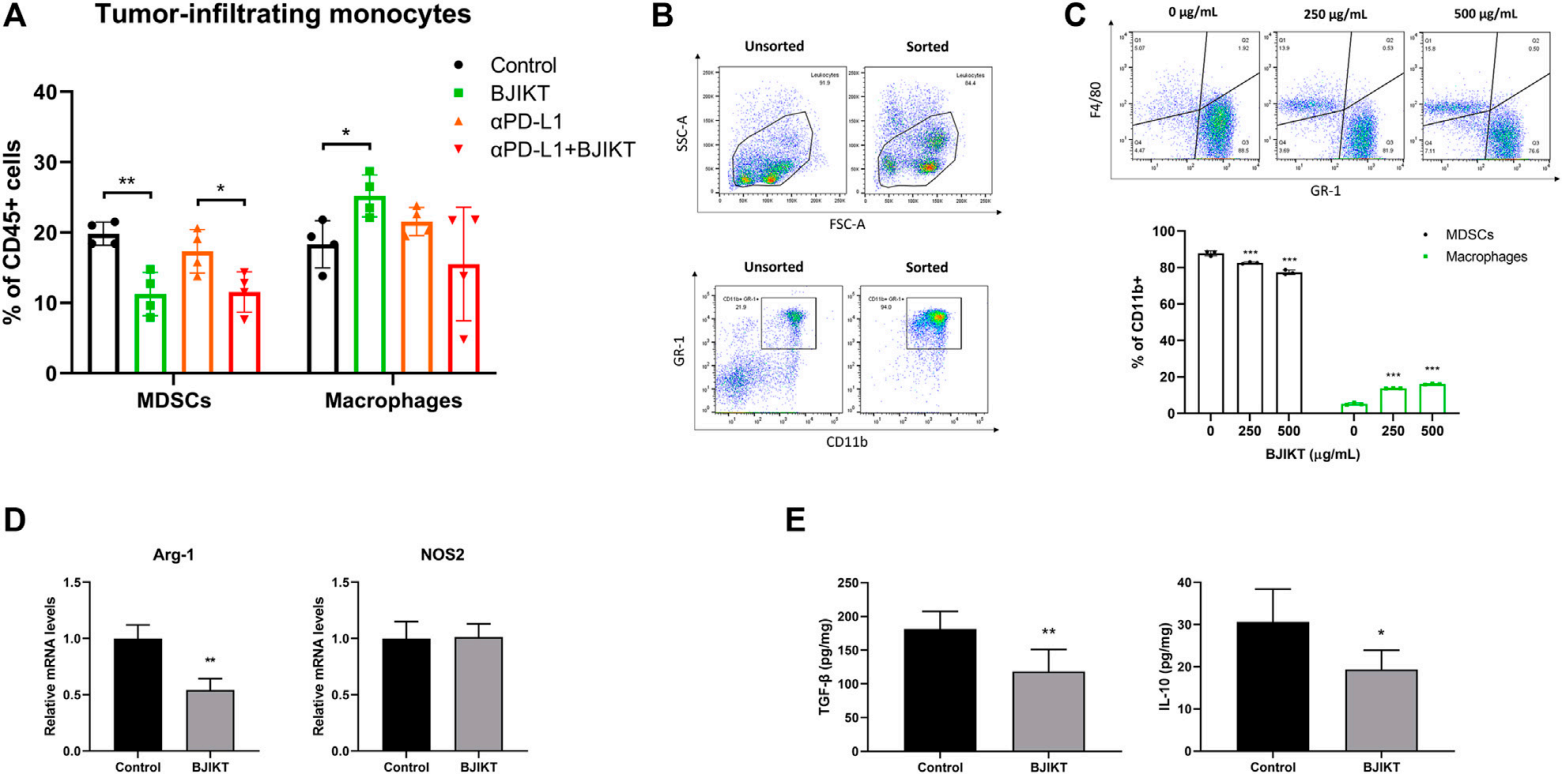
BJIKT regulated antitumor immunity by attenuating MDSCs and promoting antitumor cytokines in the tumor microenvironment. **(A)** The subsets of tumor-infiltrating monocytes, including MDSCs (CD11b^+^GR-1^+^) and macrophages (CD11b^+^F4/80^+^) in tumor tissues was confirmed using flow cytometry. *p < 0.05 and **p < 0.01, compared between each group. **(B)** The purity of MDSCs. MDSCs were purified from splenocytes isolated from the spleen of MC38 tumor-bearing mice. The sorted MDSCs were confirmed using flow cytometry. **(C)** The effect of BJIKT on MDSC differentiation *in vitro*. MDSCs were treated with BJIKT for 36 h. The subsets of MDSCs and macrophages were analyzed by flow cytometry. **(D)** Effect of BJIKT on the mRNA expression of nitric oxide synthase 2 (NOS2) and arginase 1 (ARG-1) by quantitative PCR. **(E)** Effect of BJIKT on levels of antitumor inflammatory cytokines, such as interleukin 10 (IL-10) and master regulator transforming growth factor β1 (TGF-β1) in tumor tissue. *p < 0.05, **p < 0.01, and ***p < 0.001, compared to the control group.

MDSCs, TAMs, and Tregs are immunosuppressive cells that strongly inhibit the activation of CTLs and T helper type 1 cells in the tumor microenvironment, contributing to the poor response to ICIs (Phuengkham et al., 2019). We reasoned that BJIKT has a regulatory function in controlling the tumor microenvironment. Thus, the subsets of tumor-infiltrating monocytes, including MDSCs (CD11b^+^GR-1^+^) and macrophages (CD11b^+^F4/80^+^), were investigated in tumor tissues. BJIKT treatment dramatically decreased the population of MDSCs, although the combination with anti-PD-L1 did not decrease further (Figure 6A). BJIKT may regulate the immune response of T-cells by regulating MDSCs. Interestingly, BJIKT increased the population of macrophages, although the combination with anti-PD-L1 did not affect the macrophage population. These results suggest that tumor-infiltrating monocytes may be differentiated into macrophages by BJIKT and have a beneficial effect on the tumor microenvironment. BJIKT had little effect on the population of tumor-infiltrating monocytes in LLC1-bearing mice (Supplementary Figure S4B). Therefore, we hypothesized that BJIKT itself might have antitumor immunity, mainly by regulating MDSCs in the MC38-bearing mouse model. Several substances have been reported to promote MDSC differentiation to macrophages. For instance, Jianpi Huayu Decoction, a formula of TCM for oncotherapy, promote the differentiation of MDSCs into macrophages and dendritic cells, finally enhancing antitumor immune responses (Xie et al., 2020). β-glucan or resiquimod treatment also induces the differentiation and maturation of splenic MDSCs into macrophages (Tian et al., 2013; Lee et al., 2014). To confirm this, MDSCs (purity = 94.0%) were successfully isolated from the spleen of MC38 tumor-bearing mice (Figure 6B) and treated with BJIKT for 36 h to investigate the effect of BJIKT on MDSC differentiation. The results showed that BJIKT dose-dependently reduced the population of MDSCs and increased the population of macrophages (Figure 6C). These results suggest that BJIKT induces the differentiation of MDSCs into macrophages. Arginase 1 (ARG-1) and nitric oxide synthase 2 (NOS2) is well-established as a hallmark of MDSC suppressive function (Cimen Bozkus et al., 2015). ARG-1 is important to the suppressive function of TAMs and MDSCs. NOS2, which is mainly expressed in MDSCs and macrophages, has the role in differentiating M1 macrophages from TAMs. Our results showed that 1.0 g/kg BJIKT administration dramatically decreased the ARG-1 expression in tumor-infiltrating MDSCs, but not significantly in NOS2 expression (Figure 6D). These results indicates that BJIKT could reduce the suppressive function of MDSCs. Next, we determined the transforming growth factor β1 (TGF-β1) and IL-10, which are frequently overexpressed in tumor microenvironment and immunosuppressive cytokines related to MDSCs, macrophages, and Tregs. ELISA was performed to determine the level of TGF-β1 and IL-10 from tumors obtained from control and 1.0 g/kg BJIKT-treated mice. As shown in Figure 6E, BJIKT treatment reduced the levels of TGF-β1 and IL-10, suggesting that BJIKT may reshape the immunosuppressive tumor microenvironment. Collectively, these results suggest that BJIKT treatment reduced MDSC recruitment and activity in the tumor microenvironment and enhanced the infiltration of T-cells by combination treatment.

### Network Pharmacology Analysis Predicts the Target Pathways of Bojungikki-Tang Regulating Tumor Microenvironment to Treat Colorectal Cancers

**FIGURE 7:**
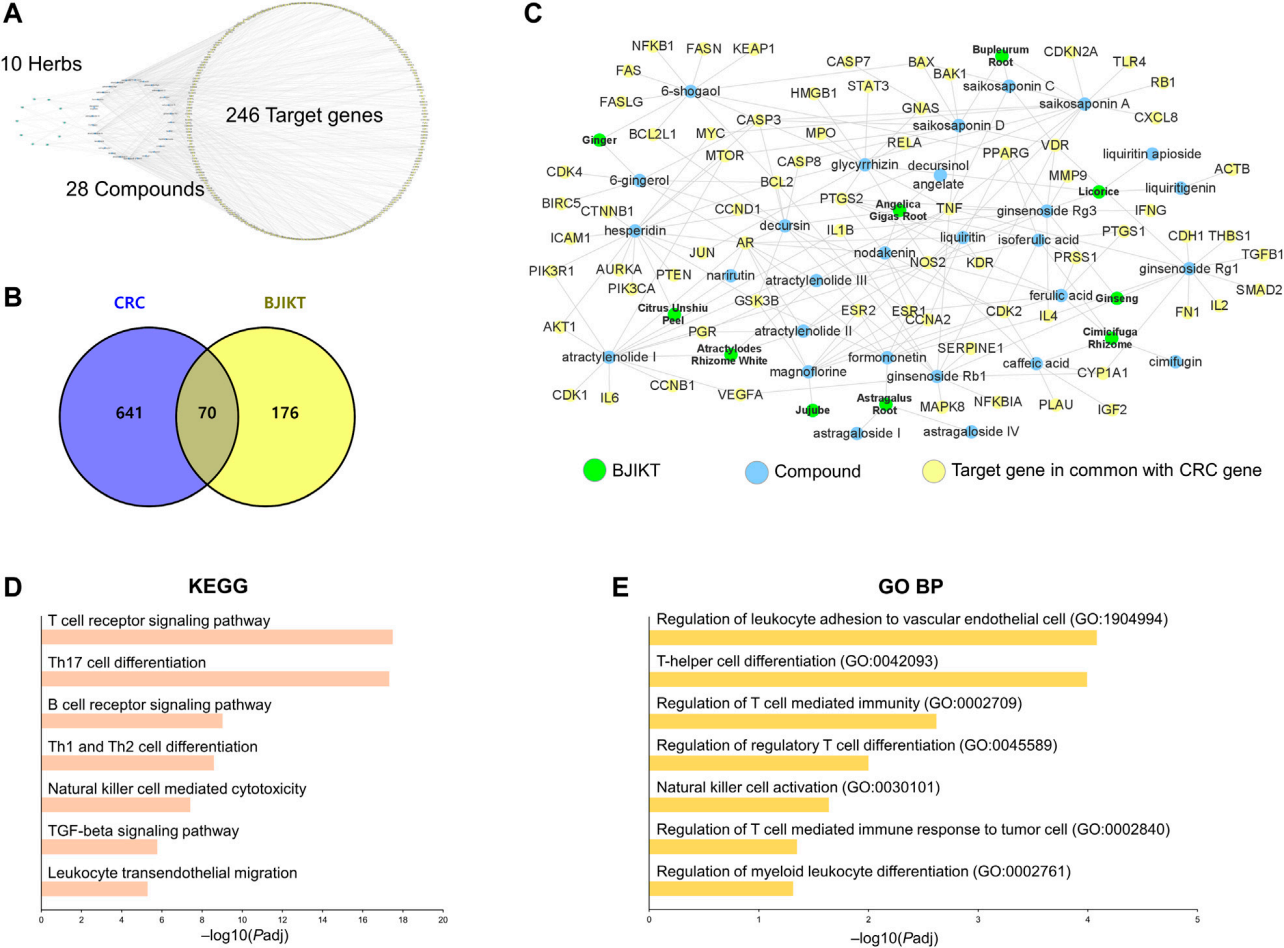
Network pharmacological analysis of BJIKT. **(A)** Network of 10 herbs, 28 major compounds, and 246 target genes of BJIKT. The potential targets of major compounds were predicted using the Encyclopedia of Traditional Chinese Medicine (ETCM) and Symptom Mapping (SymMap) databases. **(B)** Venn diagram for 711 CRC-related genes and 246 target genes of BJIKT. The 70 genes were common. **(C)** Reconstructed BJIKT network with the common 70 target genes. **(D,E)** Pathway enrichment analysis for the common 70 target genes of BJIKT with the Kyoto Encyclopedia of Genes and Genomes (KEGG) **(D)** and Gene Ontology Biological Process (GO BP) **(E)** gene sets.

The introduction of network pharmacology provides a chance to unravel the potential multiple-level actions of multiple components (Li & Zhang, 2013). To investigate the components of BJIKT in the treatment of CRC, we performed a network pharmacological analysis of BJIKT. We explored the therapeutic target proteins from the 28 major compounds detected in 10 herbs of BJIKT from two recent herbal medicine databases, ETCM and SymMap. A total of 246 genes were identified as potential BJIKT-related targets (Figure 7A). To further elucidate the multiple effects of BJIKT on CRC, 711 CRC-associated genes were extracted from GeneCards, and 70 of these genes overlapped with the putative targets of BJIKT (Figure 7B). Network interactions were established to understand the possible targets and their relationships (Figure 7C). Pathway enrichment analysis with KEGG and GO BP gene sets of the common 70 target genes showed that pathways involved in the tumor microenvironment were enriched, such as T cell-associated immunity, NK cell activation, and myeloid differentiation (Figures 7D,E). The common targets were also enriched in the TGF-β signaling pathway (Supplementary Figure S5), NK cell-mediated cytotoxicity pathway (Supplementary Figure S6), and leukocyte transendothelial migration pathway (Supplementary Figure S7). These outcomes indicate that BJIKT may modulate immunosuppression and CRC progression via multiple targets.

## Discussion

Over the past few years, a better understanding of the complicated relationships between cancer cells and the immune network system in the tumor microenvironment has led us to develop new ICIs as immunotherapeutic approaches (Tan et al., 2020). Tumor cells express PD-L1 on their surface to escape the immune response by cytotoxic T-cells. PD-L1 directly binds to PD-1, which is located on the surface of exhausted or activated T-cells, resulting in the inhibition of antitumor immune responses (Robert, 2020). Thus, anti-PD-L1 monoclonal antibodies (mAbs), such as atezolizumab, and anti-PD-1 mAbs, such as pembrolizumab and nivolumab, are effective against many cancer types and show a favorable 5-year survival rate for cancer patients. Unfortunately, however, ICIs have considerable drawbacks in many solid tumors, including low efficiency to immunotherapy (Park et al., 2018). Furthermore, ICIs have shown little clinical benefit in patients with CRC. Therefore, many effectors have been developed to enhance the tumor responses of PD-L1 and PD-1 antibodies in CRC (Huyghe et al., 2020). ICIs combined with other therapies have recently attracted much attention because they exert synergistic mechanical properties (Chen et al., 2021). Chemotherapy and targeted therapy drugs release tumor-associated antigens from destroying cancer cells, stimulating a strong immune response. Additionally, these drugs suppress immunosuppressive cells, including MDSCs and Tregs (Murciano-Goroff et al., 2020). Because of these potentials, combinations of ICIs and these drugs have been recently assessed in cancer patients. These findings suggest a new possibility of TCM as an immune-modulating drug for combination with ICIs. The TCM formula usually possesses multi-target tumor suppressive effects and may have potent immune regulatory capability in the tumor microenvironment. BJIKT inhibits tumor progression, relieves immunosuppression, and improves the quality of life of cancer patients (Cho et al., 2004; Jeong et al., 2010). Many studies have also demonstrated that BJIKT enhances its anticancer activity in combination with chemotherapy (Volate et al., 2009; Yu et al., 2017). Therefore, our study provides a rationale for combinatorial treatment using ICIs and herbal medicines.

The tumor microenvironment is dependent on the balance between the immune mediators that suppress or promote tumor progression. MDSCs, TAMs, and Tregs promote tumor progression, whereas CD4^+^ T-cells, CD8^+^ T-cells, NK cells, and dendritic cells promote tumor destruction. Although many studies are now exploring the targets of these immune cells, cytotoxic CD8^+^ T-cells are the most important effectors of the antitumor immune response (Raskov et al., 2021). CTLs directly destroy cancer cells by releasing cytotoxic granules, such as GrB, and secrete cytokines, primarily IFN-γ and TNF-α, which possess antitumor effects (Farhood et al., 2019). However, immunotherapy is ineffective in patients with solid tumors that are not infiltrated by T-cells with a non-inflamed tumor microenvironment (Heinhuis et al., 2019). Therefore, novel strategies that convert non-inflamed tumors into inflamed ones are being developed to find new targets or drugs to improve tumor immunity. PD-1/PD-L1 blockade, together with an inflamed tumor microenvironment, facilitates the infiltration of effector T cell into solid tumors (Duan et al., 2020). TCM is widely applied in the treatment and prevention of immune-related diseases. Bioactive components in TCM can improve the tumor microenvironment, consequently activating T-cells. In this study, we hypothesized that the targets of BJIKT might be associated with the antitumor immune response. We demonstrated that BJIKT suppressed tumor growth with an anti-PD-L1 antibody and significantly increased the population of CTLs and their cytotoxicity in the MC38 CRC-bearing mouse model. In our results, BJIKT slightly reduced tumor growth and did not kill tumor cells directly. Specifically, the spleen index decreased after BJIKT administration, implying that BJIKT regulates the immunological function of spleen. The spleen is a crucial immune organ in the antitumor immune response. Splenic T-cells and NK cells are the effector cells for cytotoxicity against tumor. However, it also plays an immunosuppressive role in the antitumor immune response. Splenic MDSCs accumulation during tumor growth gradually becomes immune negative and inhibits the function of T-cells in tumor-bearing mice (Li et al., 2016). Although the dynamics of the spleen during tumor progression remains incompletely understood, the decreased spleen index may be associated with the decreased population of MDSCs by BJIKT treatment, thereby restoring antitumor immunity initiated by T-cells.

Combination therapies with ICIs have been given more attention in recent years in the respect that they enhance the therapeutic response and decrease side effects during cancer treatment. To investigate the combinational effect of BJIKT with ICIs, we selected 10 mg/kg anti-PD-L1 antibody, which is a well-optimized dose in animal studies. The PD-L1 antibody injection of higher doses than 10 mg/kg may induce immune-related adverse effects or severe hypersensitivity reactions (Kim et al., 2020). In our results, combination treatment with BJIKT and anti-PD-L1 antibody enhanced the antitumor effect by promoting the immune responses of CD8^+^ T-cells. In addition, BJIKT did not interrupt PD-1/PD-L1 binding, suggesting that the antitumor effect of anti-PD-L1 was maximized by BJIKT regardless of PD-L1 status. Moreover, combination treatment of BJIKT and anti-PD-L1 antibody showed more meaningful results in the immunocompetent model than in the poorly immunogenic model, suggesting that the role of BJIKT may be significantly associated with the immune response in the tumor microenvironment. The combination therapy could elevate IFN-γ and TNF-α and decrease IL-17A in the serum of the tumor-bearing mice, which may contribute to the increase of TILs and the antitumor immunity. Interestingly, IL-17A has been known to increase the immunosuppressive activity of Tregs and promote the recruitment of MDSCs in cancer (Liu et al., 2021).

Although MDSCs are normally produced and present in the bone marrow, they are expanded in the blood, tumor, and spleen of tumor-bearing mice (Katayama et al., 2018). In tumor tissues, MDSCs comprise tumor-infiltrating immature myeloid cells that play an important role in tumor immune escape by suppressing CTL proliferation and driving Treg induction (Ugel et al., 2015). Therefore, targeting MDSCs is an important therapeutic strategy for immune regulation to facilitate cancer progression and relapse. Inhibition of activation, differentiation into maturation, and apoptosis induction were effective in targeting MDSCs in cancer therapy. We found that BJIKT decreased the population of MDSCs but increased the number of macrophages. The plasticity of MDSCs and their differentiation into macrophages had been demonstrated (Tcyganov et al., 2018). Here, we considered that BJIKT might promote the differentiation of MDSCs into macrophages. Although the combination treatment did not increase the macrophage population, this phenomenon is due to the increased population of CD3^+^ T-cells in the combination group. In addition, BJIKT did not alter the population of CD3^+^ T-cells, whereas the combination treatment increased them. There exists the complexity of the tumor microenvironment when both BJIKT and anti-PD-L1 were treated. However, this action of BJIKT may have the potential to convert a non-inflamed tumor microenvironment into an inflamed tumor microenvironment and activate the function of T-cells, promoting the effect of anti-PD-L1 antibodies. ARG-1 and NOS2 use *L*-arginine as a substrate and deplete *L*-arginine from the tumor microenvironment, producing nitric oxide and ROS, mediating T cell suppression (Raber et al., 2012). We found that BJIKT decreased the population of MDSCs and downregulated ARG-1 expression, which is associated with MDSC and M2-like macrophages in the tumor microenvironment, suggesting that BJIKT may enhance the immune response by regulating MDSCs, but may not have an effect on the population of M2-like macrophages. Although we did not confirm the subtype of macrophages in this model, M2-like macrophages appear to be a major type of macrophages in tumor microenvironment. Further studies are needed to explore the effect of BJIKT on the intratumoral differentiation of MDSCs and their roles in immune response of ICIs for CRC. In addition, our results indicated that BJIKT suppressed IL-10 and TGF-β1, suggesting that BJIKT improved antitumor immunity by suppressing the expansion of Tregs and boosting the T cell response by producing IL-10 and TGF-β. We also expected that BJIKT plays multiple roles by regulating multiple pathways as shown in the network pharmacological analysis of BJIKT. In this study, we have not yet investigated the detailed signaling pathways on MDSCs-mediated immune suppression by BJIKT, further studies will be continued to clarify the relationship between splenic MDSCs, tumor MDSCs, and their regulation of TILs.

BJIKT is composed of ten medicinal herbs, *Astragalus membranaceus* (Fisch.) Bunge, *Atractylodes macrocephala* Koidz., *Panax ginseng* C. A. Mey., *Angelica gigas* Nakai, *Bupleurum falcatum* L., *Ziziphus jujuba* var. *inermis* (Bunge) Rehder, *Citrus unshiu* Marcow., *Glycyrrhiza glabra* L., *Cimicifuga heracleifolia* Kom., and *Zingiber officinale* Roscoe. LC-MS/MS analysis was performed for the qualitative analysis of the chemical compounds of BJIKT. Twenty-eight compounds were selected from the literature review and quantified using the corresponding reference compound. Glycyrrhizin, one of the main components, regulates antitumor immunity by attenuating Tregs and MDSCs (Juin et al., 2020). Another component, hesperidin, may restore antitumor immunity in T helper cells (Yamamoto et al., 2021). Furthermore, network pharmacological analysis using 70 BJIKT-associated CRC genes suggests that BJIKT is closely related to the regulation of immune responses, T-cell-associated immunity, NK cell activation, and myeloid differentiation through multiple targets. A long-lasting immune response in cancer patients can occur by differentiation of naïve T-cells upon antigen recognition into different memory cell lineages: T_CM_ and T_EM_ (Vahidi et al., 2020). Although BJIKT increased the population of T_EM_ and NK cells, more experiments with each component are needed to explore the effect of BJIKT on the systemic inflammatory response leading to tumor response.

There are some limitations in this study. Our results showed that 1.0 g/kg BJIKT inhibited the tumor growth and synergizes with anti-PD-L1 antibody. The dose of BJIKT used in animal study was much higher than the dose given in a consensus document (Heinrich et al., 2020). However, several safety evaluation studies demonstrated that BJIKT did not induce toxic effects at a dose level up to 2.0 g/kg in animal models (Yoo et al., 2017; Jeon et al., 2020). In addition, this is the concentration of mixed herbal medicines, not the main herbal medicines or components. Therefore, further studies are needed to explore the main herbal medicines and compounds of BJIKT exerting the pharmacological effects and optimize the dosage of BJIKT. Nevertheless, our findings provide the first evidence of BJIKT’s potential as a combination treatment with anti-PD-L1 for cancer treatment.

## Conclusion

The present study demonstrated that BJIKT combined with anti-PD-L1 antibody enhanced the infiltration of CD8^+^ T-cells and restored their function, which dramatically inhibited tumor growth in the MC38 CRC model. Our present findings indicate that BJIKT regulates MDSCs and immune-related factors in the tumor microenvironment. This study provided preliminary evidence for the combination treatment of BJIKT and anti-PD-L1 antibody for a better response to ICIs. Moreover, we suggest the possibility that BJIKT has the advantages of multi-component and multi-target in the tumor microenvironment. Although the complex immune mechanisms of BJIKT involved in MDSCs and their regulation of TILs are needed to further explore, the synergistic antitumor effect of BJIKT and anti-PD-L1 antibody offers a reasonable strategy for future clinical combinatorial application to overcome the low response in patients with CRC.

## Data Availability

The raw data supporting the conclusion of this article will be made available by the authors, without undue reservation.
