# Supplementary material for: Bojungikki-Tang Improves Response to PD-L1 Immunotherapy by Regulating the Tumor Microenvironment in MC38 Tumor-Bearing Mice
Source: Front Pharmacol. 2022 Jul 6;13:901563. doi: 10.3389/fphar.2022.901563 (PMC9300825; doi:10.3389/fphar.2022.901563)
Supplement: Supplementary file 1 [file DataSheet1.docx]

**Bojungikki-Tang Improves Response to PD-L1 Immunotherapy by Regulating the Tumor Microenvironment in MC38 Tumor-Bearing Mice**

*Jaemoo Chun^1^, Sang-Min Park^2,3^, Jin-Mu Yi^1^, In Jin Ha^4^, Han Na Kang^1^, Mi-Kyung Jeong^1^**

**Affiliation**

^1^ *KM Convergence Research Division, Korea Institute of Oriental Medicine, Daejeon, South Korea*

^2^ *KM Data Division, Korea Institute of Oriental Medicine, Daejeon, South Korea*

^3^ *College of Pharmacy, Chungnam National University, Daejeon, South Korea*

^4^ *Korean Medicine Clinical Trial Center (K-CTC), Korean Medicine Hospital, Kyung Hee University, Seoul, South Korea*

* Correspondence: Mi-Kyung Jeong, K.M.D., Ph.D.

KM Convergence Research Division, Korea Institute of Oriental Medicine, 1672 Yuseong-daero, Yuseong-gu, Daejeon, 34054, South Korea

Tel: +82-42-868-9475

Fax: +82-42-868-9299

*E-mail address:* [oiny2000@kiom.re.kr](mailto:oiny2000@kiom.re.kr)


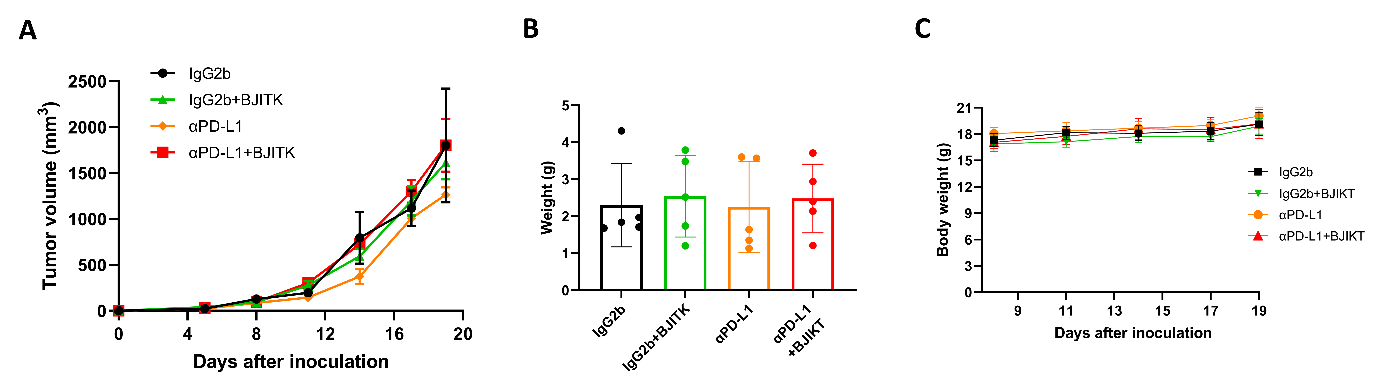


**Supplementary Fig. 1.** LLC1 cells were injected subcutaneously into C57BL/6 mice. Mice were divided into four groups (n = 5, each group) as follows: control mice, mice with BJIKT, mice with PD-L1 antibody, and mice with combination treatment (BJIKT and PD-L1 antibody) and administered BJIKT (1.0 g/kg) orally and/or anti-PD-L1 (10 mg/kg) 3 times a week. **(A)** Tumor volume changes in LLC1-bearing mice. **(B)** LLC1 tumors were weighed. **(C)** Body weight changes of LLC1-bearing mice.


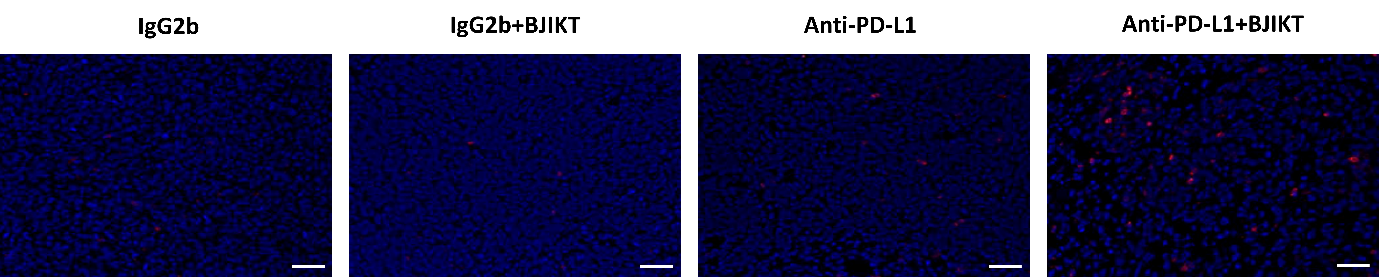


**Supplementary Fig. 2.** Representative images of CD8+ T cells immunostaining in MC38 tumor tissues. Scale bar represents 50 μm.


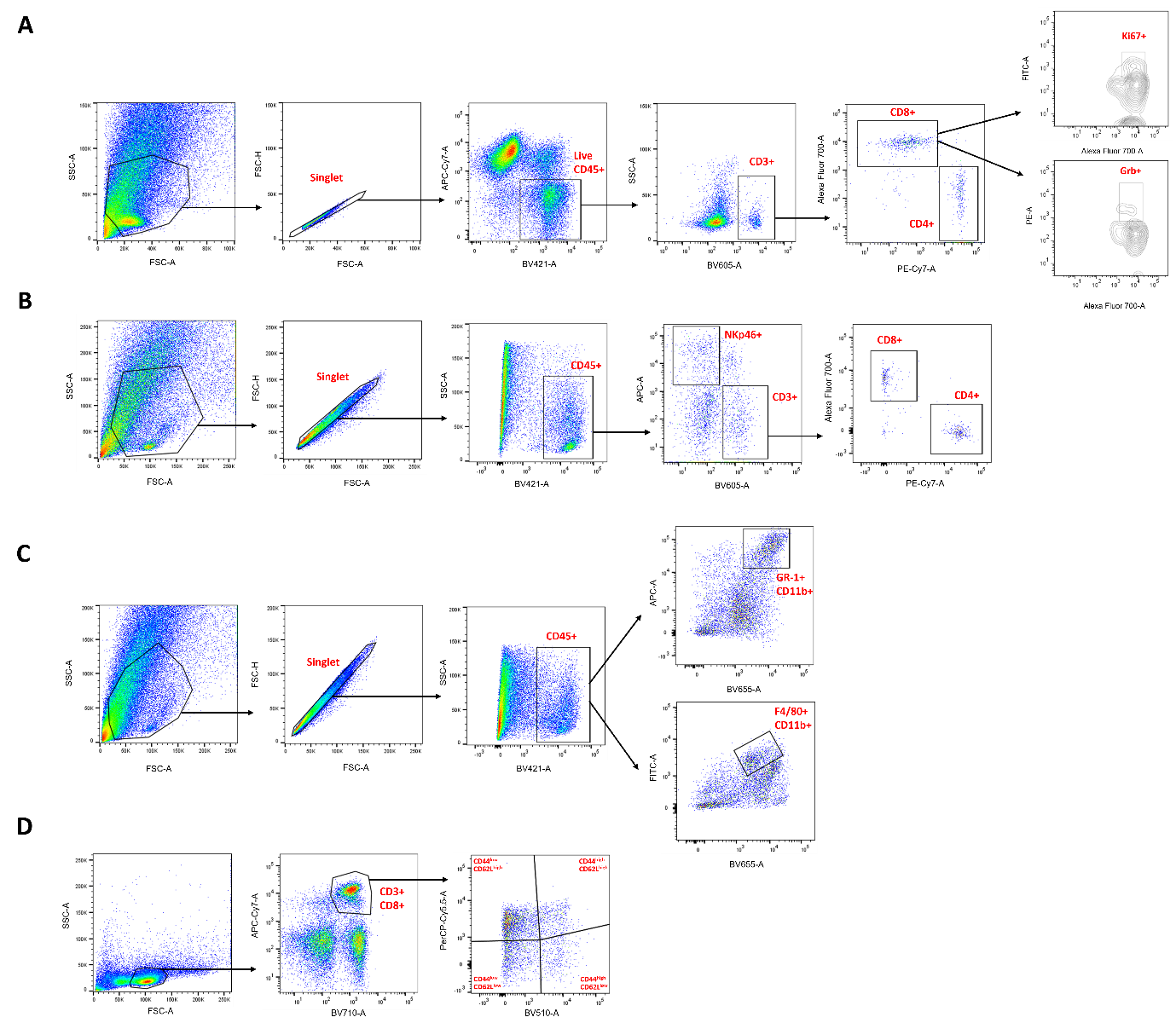


**Supplementary Fig. 3.** Gating scheme for flow cytometric analysis of immune cell populations. **(A)** Flow cytometric analysis of Ki-67-expressing and granzyme B-expressing CD8+ T cells. **(B)** Flow cytometric analysis of tumor-infiltrating lymphocytes, including T cells and NK cells. **(C)** Flow cytometric analysis of tumor-infiltrating monocytes, including MDSCs and macrophages. **(D)** Flow cytometric analysis of tumor-draining lymph node, including CD8+ T cells.


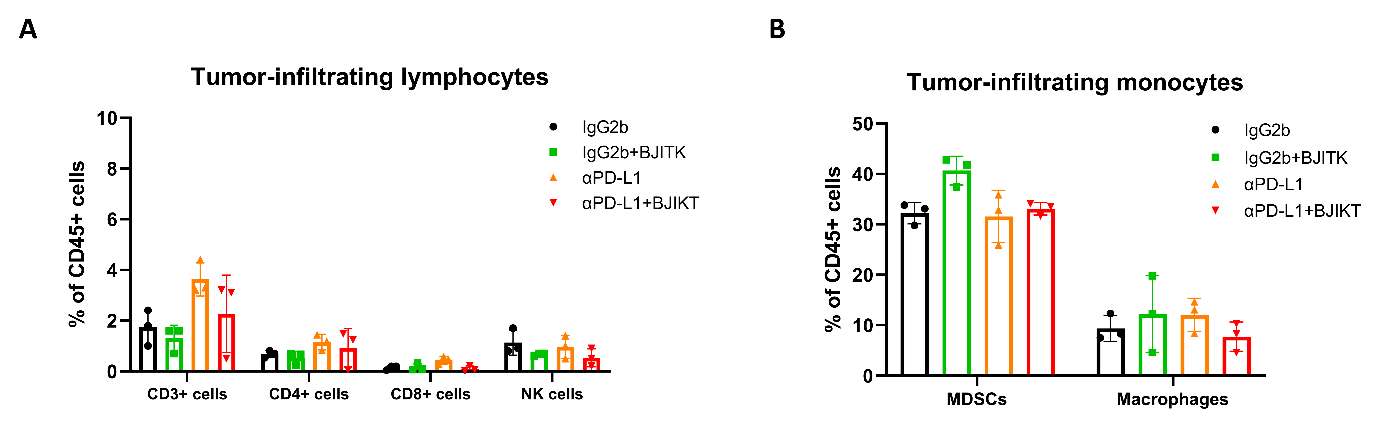


**Supplementary Fig. 4.** The immune population in LLC-bearing mice. **(A)** Tumor-infiltrating lymphocytes. **(B)** Tumor-infiltrating monocytes.


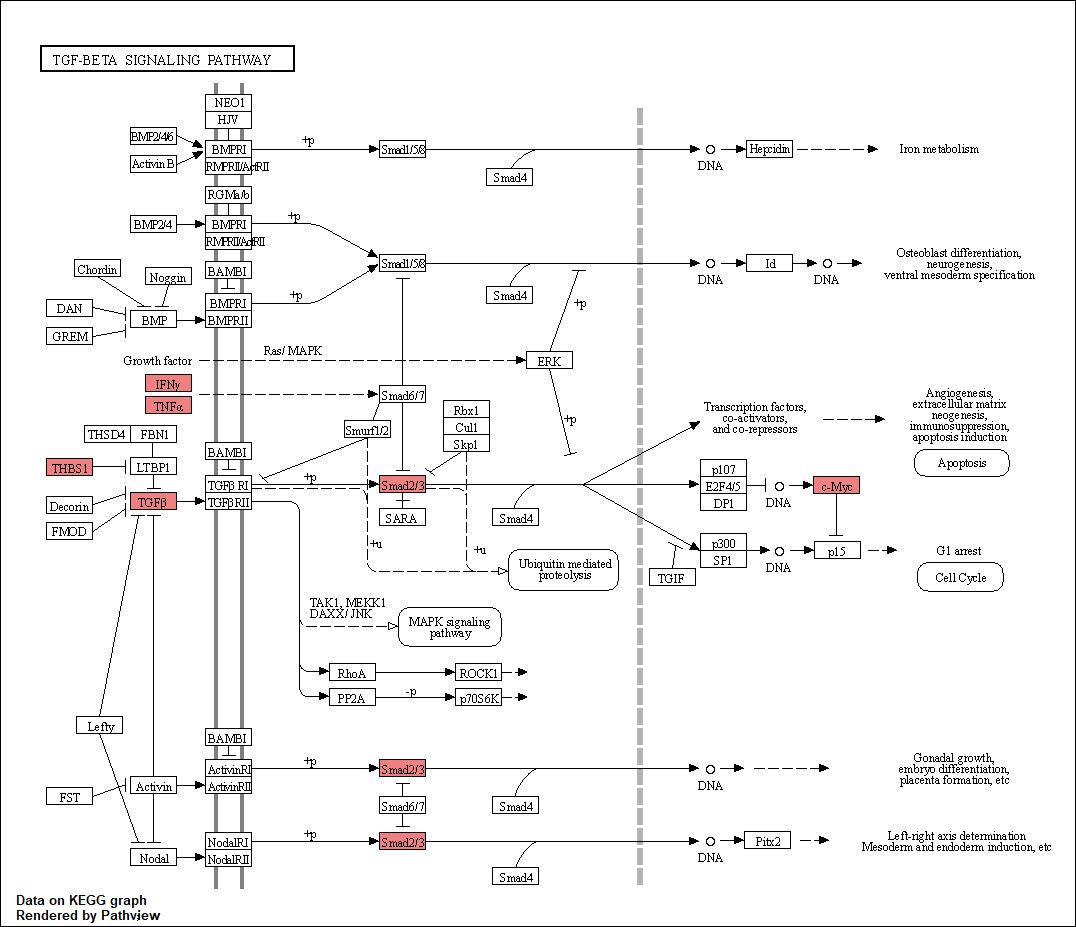


**Supplementary Fig. 5.** Distribution of the common targets between BJIKT and CRC in TGF-β signaling pathway. The common targets are denoted as red color.


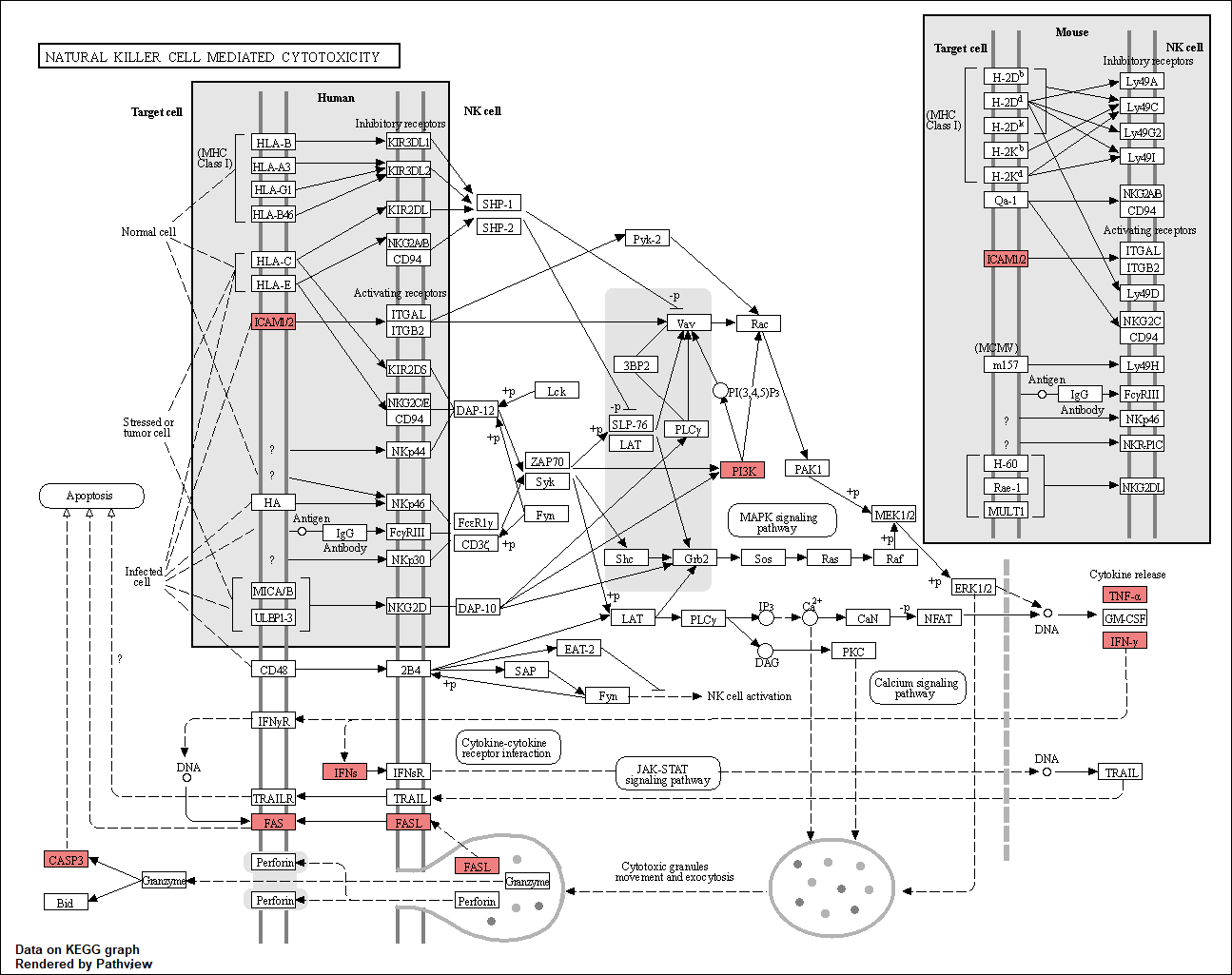


**Supplementary Fig. 6.** Distribution of the common targets between BJIKT and CRC in NK cell-mediated cytotoxicity pathway. The common targets are denoted as red color.


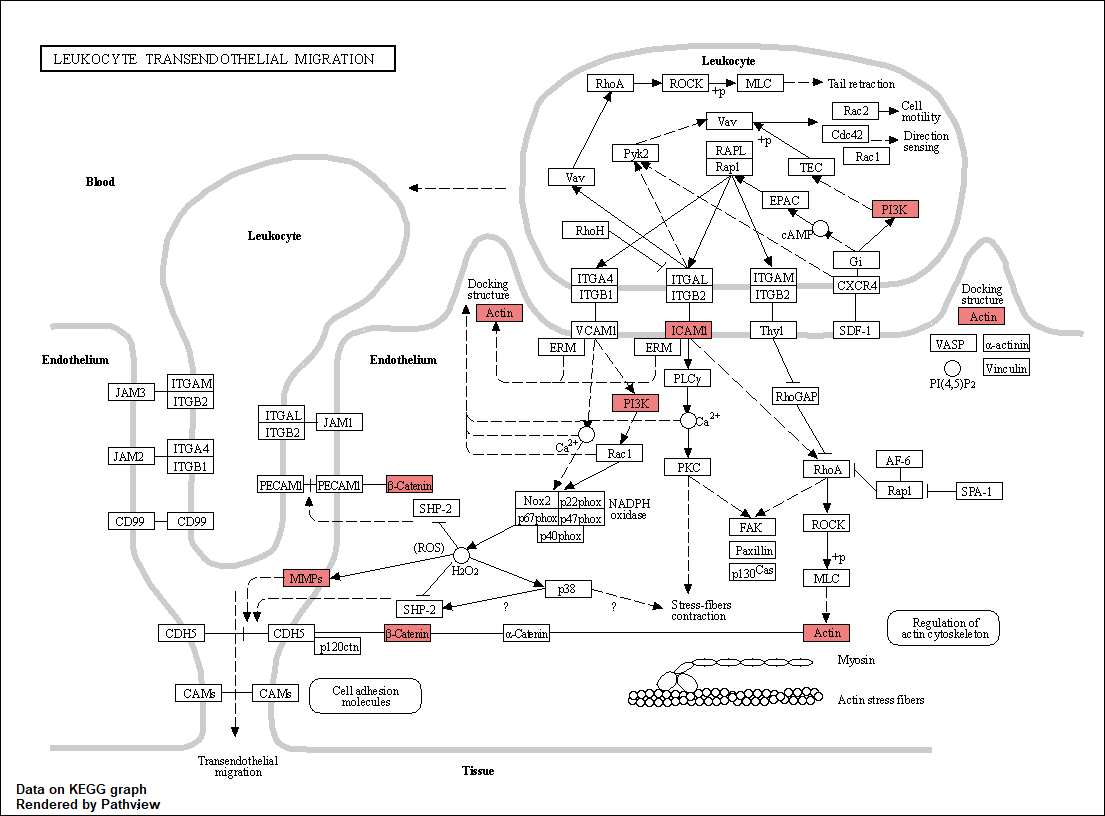


**Supplementary Fig. 7.** Distribution of the common targets between BJIKT and CRC in leukocyte trans-endothelial migration pathway. The common targets are denoted as red color.
